# YjbH mediates the oxidative stress response and infection by regulating SpxA1 and the phosphoenolpyruvate-carbohydrate phosphotransferase system (PTS) in *Listeria monocytogenes*

**DOI:** 10.1080/19490976.2021.1884517

**Published:** 2021-02-12

**Authors:** Changyong Cheng, Xiao Han, Jiali Xu, Jing Sun, Kang Li, Yue Han, Mianmian Chen, Houhui Song

**Affiliations:** College of Animal Science and Technology & College of Veterinary Medicine of Zhejiang A&F University, Key Laboratory of Applied Technology on Green-Eco-Healthy Animal Husbandry of Zhejiang Province, China-Australia Joint Laboratory for Animal Health Big Data Analytics, Zhejiang Provincial Engineering Laboratory for Animal Health Inspection & Internet Technology, Hangzhou, Zhejiang, P. R. China

**Keywords:** *Listeria*, oxidative stress, thioredoxin, PTS, bacterial infection

## Abstract

The foodborne pathogen *Listeria monocytogen*es relies on its ability to fine-tune the expression of virulence factors and stress regulators in response to rapidly changing environments. Here, we reveal that YjbH, a putative thioredoxin family oxidoreductase, plays a pivotal role in bacterial adaption to oxidative stress and host infection. YjbH directly interacts with SpxA1, an ArsC family oxidative stress response regulator, and the deletion of YjbH compromised the oxidative stress tolerance of *L. monocytogenes*. Also, YjbH is required for the bacterial spread in host cells and proliferation in mouse organs, thereby contributing to virulence. Transcriptomic analysis of strains treated with Cd^2+^ revealed that most virulence genes and phosphoenolpyruvate-carbohydrate phosphotransferase system (PTS) genes were significantly downregulated in the absence of YjbH. However, YjbH inhibits PrfA expression when bacteria were grown in the media, suggesting that YjbH participates in regulating the virulence genes via a complicated regulatory network involving PrfA and PTS. Collectively, these findings provide a valuable model for clarifying the roles of thioredoxins from foodborne pathogens regarding improving survival in the external environment and, more importantly, successfully establishing infection within the host.

## Introduction

*Listeria monocytogenes* is an intracellular facultative bacterial pathogen that can cause serious infections leading to a high mortality rate in immunocompromised individuals.^1^ This pathogen is well-adapted to various stress environments and can replicate in animal cells and the external environment.^2^ The move from high-stress environments to the cytosol requires the interplay of *L. monocytogenes* factors that promote survival in the gut, bacterial invasion, and phagosomal escape, which is followed by replication and movement within the cytosol and spread to adjacent cells.^2^ During the adaption phase, the most common stress encountered by the bacteria is oxidative stress, which can damage cellular components.^3^ Bacterial pathogens have evolved sophisticated mechanisms to sense and adapt to oxygen-rich environments by producing catalases, thioredoxins, peroxiredoxins, and superoxide dismutases, which neutralize harmful oxidants before they cause damage to cellular components.^4,5^ *L. monocytogenes* is phagocytosed by macrophages, in which it transiently resides within the oxidizing environment of the vacuole.^6^ After internalization, the secreted pore-forming toxin listeriolysin O (LLO) and two phospholipases (PlcA and PlcB) rapidly mediate escape from the oxidizing phagosome into the highly reducing cytosol,^7-9^ which facilitates survival, intracellular replication, and eventually spreads into neighboring cells.^10^ Therefore, *L. monocytogenes* is an excellent model system for studying bacterial adaptive responses to redox changes during host infection.^11,12^

Bacteria maintain the cytosolic redox status largely by using redox-regulating thiol molecules such as glutathione and the dicysteine proteins thioredoxin and glutaredoxin.^13^ At the expense of NADPH, glutathione is maintained in a reduced state by glutathione reductase, and thioredoxin is kept reduced by thioredoxin reductase.^14^ Many Gram-positive bacteria mainly employ low-molecular-weight thiols, thioredoxin, and alternative thioredoxin-based enzymes as antioxidant systems.^15,16^ The thioredoxin family contains a common structural fold (the Trx domain) and is involved in cellular defense against oxidative stress caused by reactive oxygen species (ROS).^17^ Thioredoxin is the major cellular disulﬁde reductase in cells, which can provide a highly reducing environment and then function as an effector to facilitate correct oxidative protein folding. In *L. monocytogenes*, PrfA, a cAMP receptor protein (Crp) family transcriptional regulator that is essential for virulence gene expression and pathogenesis,^18^ is exclusively activated in the cytosol of host cells. The glutathione generated by bacteria or derived from host cells is the essential small-molecule cofactor of PrfA. Glutathione allosterically binds to PrfA, thereby increasing its activity regarding inducing target genes.^19,20^

Based on a search for the Cys-X-X-Cys motif (a hallmark of thioredoxins for sensing oxidative stress), 14 homologs of the thioredoxin family members have been predicted in the *L. monocytogenes* EGD-e genome.^16,21^ Our present research focuses on these proteins, as we aim to understand their biological functions and underlying mechanisms. Previously, we determined that *L. monocytogenes* thioredoxin A (encoded by *lmo1233*), a vital cellular reductase, is essential for maintaining a highly reducing environment in the bacterial cytosol, which provides favorable conditions for correct protein folding, and therefore contributes to the bacterial antioxidant system and virulence.^22^ Additionally, Reniere and colleagues recently cleverly used a transposon screening strategy and identified a putative thioredoxin, YjbH, in *L. monocytogenes* that was found to be required for translational activation of an essential determinant of *L. monocytogenes* pathogenesis (ActA) during infection.^11^ However, the precise mechanisms underlying YjbH-dependent regulation of *L. monocytogenes* virulence and the redox stress response have not yet been elucidated. In *Bacillus subtilis*, YjbH is thought to function in coordination with Spx,^23-25^ an arsenate reductase (ArsC) family transcriptional regulator that activates and represses transcription in response to oxidative stress *via* direct interaction with the α subunit of RNA polymerase (RNAP).^26-30^ Under nonstress conditions, the soluble YjbH adaptor protein interacts with the Spx C-terminus, resulting in the rapid degradation of Spx by the ATP-dependent protease ClpXP complex. In response to stress, an aggregation of YjbH becomes surface-exposed, leading to a rapid formation of YjbH self-aggregates.^31^ YjbH thereby loses its ability to target Spx for degradation, resulting in Spx accumulation. This enables activation of >140 genes and operons to help reestablish the cytoplasmic thiol-disulfide redox balance and repair the stress-related damage.^12,28^ *L. monocytogenes* encodes two Spx paralogs, SpxA1 and SpxA2, and SpxA1 but not SpxA2 is required for resistance to oxidative stress and pathogenesis, suggesting a critical role for SpxA1 in redox homeostasis and virulence.^5,11^ However, the interactive relationships and underlying mechanisms of SpxA1-YjbH remain unclear.

Here, we further elucidated the regulatory roles of YjbH in response to redox stress in *L. monocytogenes*. Importantly, we demonstrated that YjbH, localized to the plasma membrane, interacts with SpxA1 and contributes to defense against oxidative stress, as well as playing an essential role in intracellular infection and pathogenesis by positively regulating the expression of most virulence factors in *Listeria* pathogenicity island 1 (LIPI-1). Surprisingly, most of the phosphoenolpyruvate–carbohydrate phosphotransferase system (PTS) genes were significantly downregulated in Δ*yjbH* compared to the wild-type strain after treatment with the oxidant Cd^2+^. Under nonstress conditions, YjbH no longer contributed to regulating the PTS genes, but PrfA expression was strongly induced in Δ*yjbH*. Collectively, these findings demonstrate that YjbH (coordinating with SpxA1) is the dominant thioredoxin family member required for the stress response and, more importantly, it is essential for pathogenesis during *L. monocytogenes* infection via the regulatory network involving PrfA and PTS.

## Results

### L. monocytogenes YjbH contributes to colony morphology and motility

**Figure 1.**
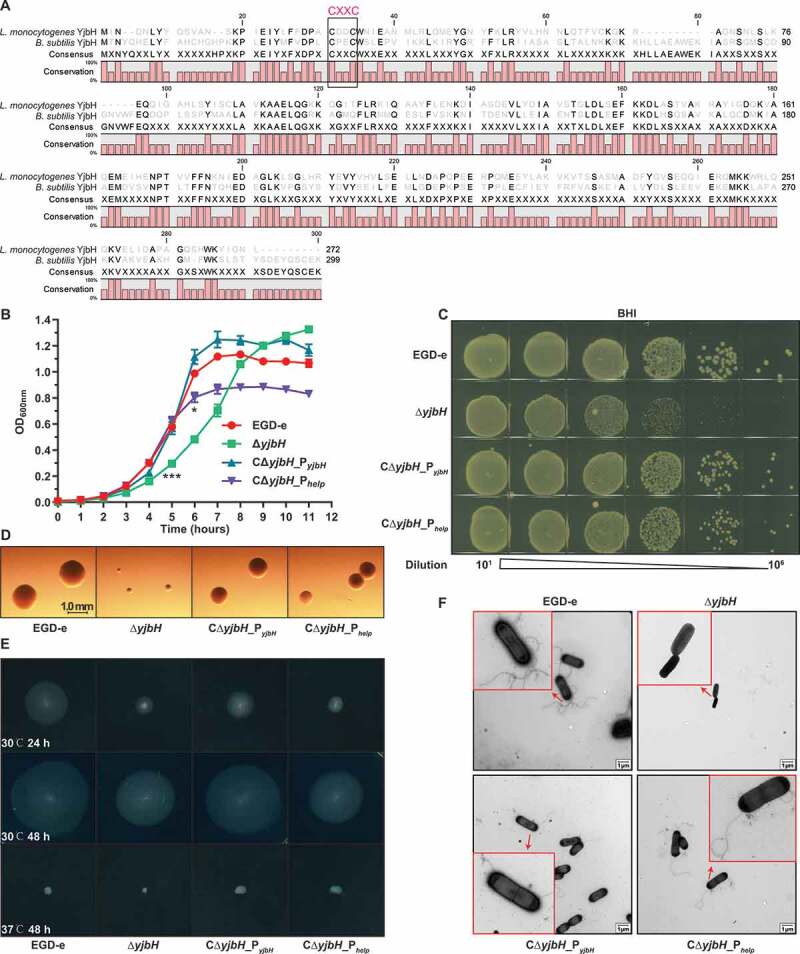
***L. monocytogenes* YjbH contributes to colony morphology and motility**. (a) Alignment of *L. monocytogenes* YjbH with the *B. subtills* homolog. The conserved CXXC motif is framed with a black line. (b-c) In vitro growth of wild-type *L. monocytogenes* EGD-e and *yjbH* mutants in BHI broth (b) or BHI agar plates (c). In (b), bacteria grown overnight were diluted (1:100) in fresh BHI broth and incubated at 37°C for 12 h. Kinetic growth at OD_600 nm_ was measured at 1-h intervals. Data are expressed as mean ± SEM of three replicates. *, *P*< .05; ***, *P*< .001. In (c), bacteria grown overnight were serially diluted, plated on BHI agar medium, and incubated at 37°C for 24 h. (d) Colony morphology observed using a stereomicroscope after growth on BHI agar plates for 24 h. (e-f) Bacterial motility assay (e) and flagellar formation observed by transmission electron microscopy (f). *L. monocytogenes* was grown on soft agar (0.25%) at 30°C or 37°C

Bioinformatic analysis indicated that *L. monocytogenes* YjbH (encoded by *lmo0964*) shares 57% amino acid similarity to the *B. subtilis* YjbH^32^ and also contains a canonical CXXC motif that is highly conserved in proteins in the thioredoxin family and is required for sensing oxidative stress (Figure 1(a)). To further explore the biological roles of YjbH, the deletion mutant Δ*yjbH* and two complemented strains (CΔ*yjbH*_P*_yjbH_*, expressing YjbH driven by its native promoter, and the YjbH-overexpression mutant CΔ*yjbH*_P*_help_*, carrying the constitutive promoter, P*_help_*) were constructed. After growing all the strains in broth at 37°C, Δ*yjbH* exhibited a significantly extended lag-phase (starting from hour 5), and CΔ*yjbH*_P*_help_* only showed a significant growth defect at the stationary phase (starting from hour 6) comparing with the wild-type and CΔ*yjbH*_P*_yjbH_* strains (Figure 1(b)). When grown in the Brain Heart Infusion (BHI) agar plates, these strains showed comparable colony-forming unit (CFU) numbers. However, the colony size of Δ*yjbH* markedly decreased compared to those of the two complemented strains which only displayed a slight decrease relative to the wild-type strain (Figure 1(c,d)). Interestingly, YjbH was found required for bacterial motility, as *yjbH* deletion compromised the swarming ability and flagellar production in *L. monocytogenes* at 30°C; this defective phenotype was rescued by complementing Δ*yjbH* with the naturally expressed YjbH (Figure 1(e,f)). Together, these data suggest that *L. monocytogenes* YjbH has a pleiotropic role regarding adaptation to external environments.

### YjbH interacts with SpxA1 in response to Cu^2+^- and Cd^2+^-induced oxidative stress

**Figure 2.**
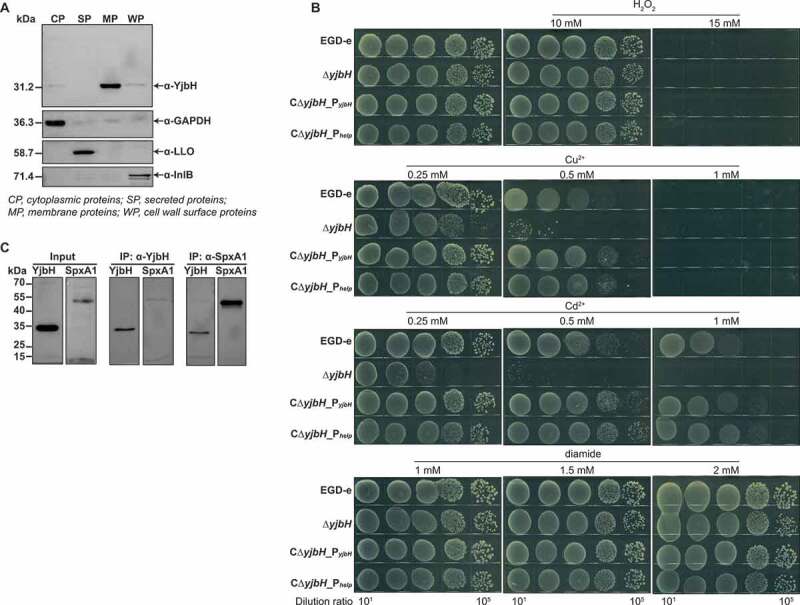
**YjbH interacts with SpxA1 in response to Cu^2+^- and Cd^2+^-induced oxidative stress**. (a) Localization of YjbH in *L. monocytogene*s. Protein fractionation was conducted, and YjbH was detected using specific polyclonal antibodies. CP, cytoplasmic proteins; SP, secreted proteins; MP, membrane proteins; WP, cell wall surface proteins. GAPDH, LLO (listeriolysin O), or InlB (internalin B) was used as the internal control for each fraction. Proteins were separated through a 12% SDS PAGE and immunoblotted with α-YjbH, α-InlB, α-LLO, or α-GAPDH antisera. (b) Survival of *L. monocytogenes* in oxidative stress conditions. Wild-type *L. monocytogenes* EGD-e and *yjbH* mutants were grown overnight, serially diluted, and then spotted onto BHI plates containing various concentrations of Cu^2+^, Cd^2+^, diamide, or H_2_O_2_ and incubated for 24–48 h at 37°C. (c) YjbH interacts with SpxA1. Coimmunoprecipitation (Co-IP) experiments were performed to detect the interaction between YjbH and SpxA1. Whole-cell lysates from *L. monocytogenes* were immunoprecipitated using anti-SpxA1 or anti-YjbH antibodies, followed by immunoblotting analysis using the indicated antibodies

YjbH homologs and other thioredoxins are responsible for bacterial resistance to oxidative stress,^14,28,29,33,34^ which prompted us to investigate the corresponding roles of YjbH in *L. monocytogenes. L. monocytogenes* YjbH was mainly localized to the plasma membrane, and a small amount of it was anchored to the cell wall (Figure 2(a)). For oxidative stress, we used three types of oxidants: metal ions (Cu^2+^ and Cd^2+^) as inducers of lipid peroxidation, H_2_O_2_ as an endogenous source of ROS, and diamide as a thiol-oxidizing agent that mimics damage due to oxygen exposure.^35,36^ Δ*yjbH* was hypersensitive to Cu^2+^- and Cd^2+^-induced oxidative stress compared to the wild-type and complemented strains (Figure 2(b)). Surprisingly, when exposed to H_2_O_2_ or diamide, Δ*yjbH* exhibited a similar sensitivity to the wild-type strain (Figure 2(b)), which is beyond our expectation that the thioredoxin family members should be responsible for resistance to H_2_O_2_-induced stress or diamide-induced stress (thiol-oxidizing stress). Regardless of the oxidative stress response for which YjbH is responsible, the phenotype of Δ*yjbH* suggested that YjbH might interact with the global regulator SpxA1 and serve as an adaptor in order to finely control the ClpXP protease-mediated degradation of SpxA1. To explore the interaction of YjbH with SpxA1 *in vivo*, co-immunoprecipitation (Co-IP) experiments were performed using YjbH-overexpressing *L. monocytogenes*. YjbH was co-immunoprecipitated with SpxA1, and in turn, SpxA1 could also be co-immunoprecipitated with YjbH (Figure 2(c)). Hence, for the first time, these findings provide strong evidence for the important role of *L. monocytogenes* YjbH in response to metal ion-induced oxidative stress as an adaptor protein that interacts with the global redox regulator, SpxA1.

### YjbH supports intracellular infection and virulence

**Figure 3.**
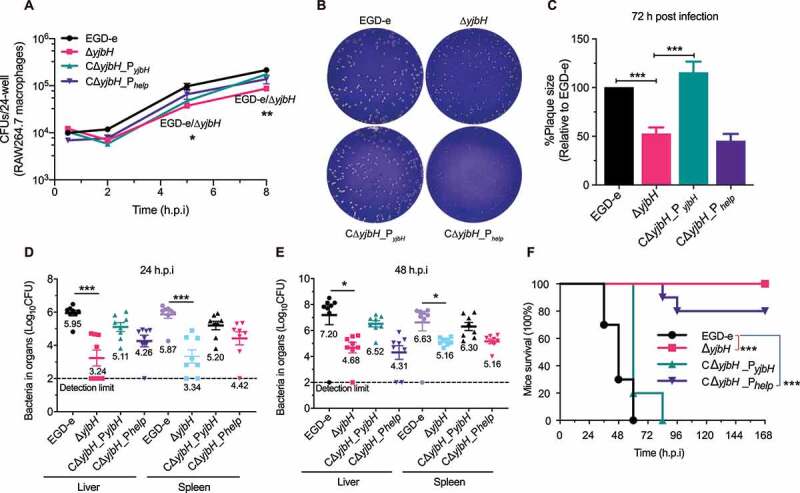
**YjbH is required for intracellular infection and virulence**. (a) Intracellular growth of wild-type *L. monocytogenes* and *yjbH* mutants in RAW264.7 macrophages. The infected macrophages were lysed at 2, 6, 12, and 18 h, and viable bacteria were serially plated on BHI plates. The number of recovered bacteria able to invade cells and survive are expressed as mean ± SEM of three replicates for each strain. (b-c) Plaque assay performed using L929 fibroblasts. The plaque size is presented as a percentage of the size associated with the wild-type strain. Data are expressed as mean ± SEM of randomly-selected plaques for each strain. (d-e) Proliferation of *L. monocytogenes* in mice organs. The wild-type and mutant strains were inoculated intraperitoneally into ICR mice at ~4 × 10^6^ CFU. Animals were euthanized at 24 (d) or 48 (e) h post-infection, and organs (livers and spleens) were recovered and homogenized. Homogenates were serially diluted and plated on BHI agar. Numbers of bacteria colonizing the organs are expressed as mean ± SEM of the log_10_CFU per organ for each group (8 mice). (f) Kaplan–Meier curve showing the survival of the ICR mice over time. Ten mice in each experimental group were injected intraperitoneally with 1 × 10^7^ CFU of *Listeria* and monitored for up to 7 days after infection. Data are presented as the percentage survival over time, and significance was determined *via* a log-rank test. ns, no significance; **, *P*< .01; ***, *P*< .001

To investigate the roles of YjbH in *L. monocytogenes* infection, murine RAW264.7 macrophages were infected with wild-type *L. monocytogenes* and Δ*yjbH*, and bacteria harvested at each time-point were plated to count the CFUs. The Δ*yjbH* displayed a slight defect in its ability to grow intracellularly at 5 and 8 h, compared to wild-type bacteria (Figure 3(a)), suggesting a minor role of YjbH to support *L. monocytogenes* grow within macrophages. In a plaque assay of cell-to-cell spread, Δ*yjbH* formed plaques that were 50% of the size of wild-type plaques and, more importantly, this defect was rescued by natural expression of *yjbH* but not by overexpression of *yjbH* (Figure 3(b,c)). To further examine the role of *yjbH* during infection of a mammalian host, mice were intraperitoneally injected with bacteria. At 24 or 48 h post-infection, the spleens and livers were harvested and homogenized, and the bacteria were incubated on BHI agar for CFU counting. Δ*yjbH*-infected mice exhibited bacterial burdens that were about 2.5 logs lower than wild-type strain-infected mice. Additionally, *yjbH* overexpression also severely attenuated virulence, whereas complementing *yjbH* using its natural promoter partially restored the virulence (Figure 3(d,e)). Furthermore, *yjbH* deletion or overexpression resulted in 100% or 80% survival, respectively, at 7 days post-infection, while wild-type or CΔ*yjbH*_P*_yjbH_* bacteria led to 100% mortality at ≤4 days post-infection (figure 3(f)). These findings demonstrate that *yjbH* deletion or overexpression *in vivo* might disturb the equilibrium of the intracellular redox potential and thus harm the pathogen’s ability to establish a successful infection.

### YjbH alters global expression profiles under oxidative stress, especially expression of the PTS and virulence genes

**Figure 4.**
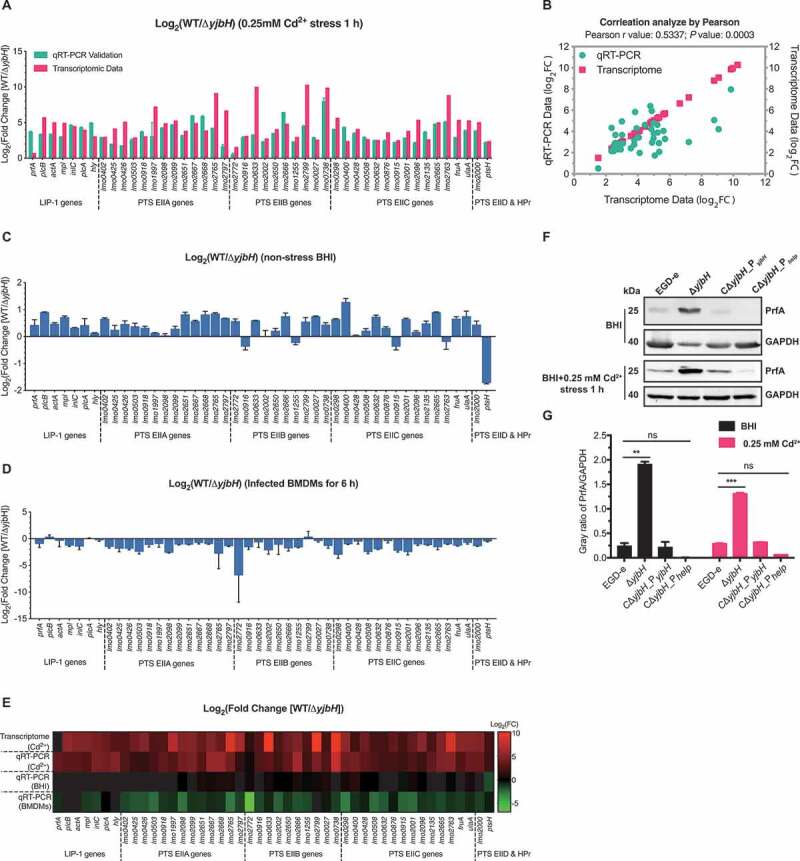
**YjbH alters global gene expression profiles, including the virulence genes and the phosphoenolpyruvate–carbohydrate phosphotransferase system (PTS) genes**. (a-b) qRT-PCR validation of the transcriptomic data for wild-type *L. monocytogenes* EGD-e and the deletion mutant Δ*yjbH* under 0.25 mM Cd^2+^ stress. *Listeria* pathogenicity island 1 (LIPI-1) genes and 39 PTS genes were selected for transcriptional assay by qRT-PCR (a), and Pearson’s correlation coefficient (r) was used to assess the consistency of differentially expressed gene (DEG) expression profiles (b). (c) Transcriptional changes of the selected DEGs under nonstress conditions. Total RNA was extracted from wild-type *L. monocytogenes* and Δ*yjbH* to the exponential phase in BHI medium and then analyzed using qRT-PCR. (d) Transcriptional changes of the PTS and virulence genes from intracellularly grown *L. monocytogenes* in BMDMs 6 hours post-infection. (e) Heatmap of the transcriptional profiles of the selected DEGs using all the data from the transcriptomic and qRT-PCR analysis of the LIPI-1 and PTS genes under nonstress or Cd^2+^ stress (0.25 mM) conditions. (f-g) YjbH controls PrfA expression. PrfA expression in wild-type *L. monocytogenes* and the *yjbH* deletion and complemented mutants were assayed by western blotting. GAPDH was used as internal control. Grayscale represents the ratio of YjbH to GAPDH. ns, no significance; **, *P*< .01; ***, *P*< .001

Whole-genome transcriptomic sequencing revealed 530 downregulated and 531 upregulated genes (fold change≥2, *P*< .05) in Δ*yjbH* after exposure to 0.25 mM Cd^2+^ for 1 h (Tables S1 and S2). The transcriptomic data have been deposited in the NCBI server (Accession No. SRP297969). Most of the differentially expressed genes (DEGs) are membrane components involved in transport. To our surprise, most of the *Listeria* virulence factors (PlcA, PlcB, ActA, Mpl, LLO, and InlC) and 56 PTS genes were significantly downregulated in Δ*yjbH* compared to the wild-type strain. These DEGs include the cytoplasmic and membrane components of PTS: enzyme I (EI), histidine-containing phosphocarrier protein (HPr), and sugar-specific enzyme II (EII domains) (Table 1). To validate the reliability of the RNA-seq results, all the *Listeria* virulence factors and 39 PTS genes from Table S1 were selected for Real-Time Quantitative Reverse Transcription PCR (qRT-PCR) analysis, and Pearson’s correlation coefficient (r) was used to assess the consistency of the DEG expression profiles. The selected DEGs showed a consistent expression pattern, with a Pearson’s r of 0.5337 (*P*= .0003) (Figure 4(a,b)), suggesting that the transcriptomic results were reliable. Next, we used qRT-PCR to compare the transcriptional changes of these genes in the wild-type and Δ*yjbH* strains under nonstress conditions, and most of them were slightly differentially transcribed (fold change<2), though *ptsH* was upregulated 3.3-fold in Δ*yjbH* (Figure 4(c)), indicating that the expression changes were far milder than those under oxidative stress. However, transcription of these genes from *L. monocytogenes* growing within the bone marrow-derived macrophages (BMDMs) was higher for the Δ*yjbH* mutant than the wild-type strain, albeit the fold-change is slight for the virulence and most PTS genes (Figure 4(d)). These results collectively indicate that YjbH may differentially control the PTS and virulence genes transcription under different conditions from environmental to host conditions, as collectively visualized in the transcriptional heatmaps (Figure 4(e)).

**Table 1.** The PTS genes that were differentially transcribed in WT and Δ*yjbH.*

PTS component	Gene	Annotation	Fold Change	Significance
			(Log_2_WT/Δ*yjbH*)	
EIIA	*lmo2765*	PTS cellobiose-specific enzyme IIA	9.08	Yes
	*lmo1997*	PTS mannose-specific enzyme IIA	7.20	Yes
	*lmo2797*	PTS mannitol-specific enzyme IIA	6.64	Yes
	*lmo2099*	PTS mannitol/fructose-specific enzyme IIA	5.23	Yes
	*lmo0426*	PTS fructose-specific enzyme IIA	5.07	Yes
	*lmo2667*	PTS galactitol-specific enzyme IIA	4.87	Yes
	*lmo2098*	PTS galactitol-specific enzyme IIA	4.85	Yes
	*lmo0425*	PTS mannitol/fructose-specific enzyme IIA	4.11	Yes
	*lmo2668*	PTS mannitol/fructose-specific enzyme IIA	3.83	Yes
	*lmo2651*	PTS mannitol-specific enzyme IIA	3.78	Yes
	*lmo0916*	PTS lactose/cellobiose-specific enzyme IIA	3.06	Yes
	*lmo0918*	PTS mannitol/fructose-specific enzyme IIA	3.02	Yes
	*lmo0402*	PTS mannitol/fructose-specific enzyme IIA	2.95	Yes
	*lmo0503*	PTS fructose-specific enzyme IIA	2.87	Yes
	*lmo0501*	PTS fructose/mannitol-specific enzyme IIA	1.67	Yes
	*lmo2697*	PTS mannose-specific enzyme IIA	1.03	Yes
	*lmo0351*	PTS mannnose-specific enzyme IIA	−2.87	Yes
	*lmo2259*	PTS beta-glucoside-specific enzyme IIA	−1.37	Yes
EIIB	*lmo2782*	PTS cellobiose-specific enzyme IIB	∞	Yes
	*lmo0633*	PTS fructose-specific enzyme IIB	∞	Yes
	*lmo2799*	PTS mannitol-specific enzyme IIBC	10.27	Yes
	*lmo0738*	PTS beta-glucoside-specific enzyme IIABC	9.85	Yes
	*lmo2666*	PTS galacticol-specific enzyme IIB	4.79	Yes
	*lmo2650*	PTS L-ascorbate-specific enzyme IIB	4.02	Yes
	*lmo1255*	PTS trehalose-specific enzyme IIBC	2.93	Yes
	*lmo2002*	PTS mannose-specific enzyme IIB	2.72	Yes
	*lmo0027*	PTS beta-glucosides specific enzyme IIABC	2.55	Yes
	*lmo0022*	PTS fructose-specific enzyme IIB	2.24	Yes
	*lmo2685*	PTS cellbiose-specific enzyme IIB	2.16	Yes
	*lmo2683*	PTS cellbiose-specific enzyme IIB	2.09	Yes
	*lmo2733*	PTS fructose-specific enzyme IIABC	2.00	Yes
	*lmo0914*	PTS cellbiose-specific enzyme IIB	1.79	Yes
	*lmo2373*	PTS beta-glucoside-specific enzyme IIB	1.65	Yes
	*lmo2772*	PTS glucose/sucrose-specific enzyme IIB	1.52	Yes
	*lmo1035*	PTS beta-glucoside-specific enzyme IIABC	1.42	Yes
EIIC	*lmo2763*	PTS cellbiose-specific enzyme IIC	8.80	Yes
	*lmo0298*	PTS beta-glucoside-specific enzyme IIC	5.62	Yes
	*lmo2096*	PTS galactitol-specific enzyme IIC	5.35	Yes
	*fruA*	PTS fructose-specific enzyme IIABC	5.33	Yes
	*ulaA*	PTS ascorbate-specific enzyme IIC	5.24	Yes
	*lmo2665*	PTS galactitol-specific enzyme IIC	4.99	Yes
	*lmo0428*	PTS fructose-specific enzyme IIC	4.06	Yes
	*lmo2001*	PTS mannose-specific enzyme IIC	3.78	Yes
	*lmo0876*	PTS lichenan-specific enzyme IIC	3.57	Yes
	*lmo0508*	PTS galactitol-specific enzyme IIC	2.61	Yes
	*lmo2135*	PTS fructose-specific enzyme IIC	2.60	Yes
	*lmo0632*	PTS fructose-specific enzyme IIC	2.49	Yes
	*lmo0915*	PTS cellbiose-specific enzyme IIC	2.48	Yes
	*lmo0400*	PTS fructose-specific enzyme IIC	2.36	Yes
	*lmo2783*	PTS cellbiose-specific enzyme IIC	2.08	Yes
	*lmo2684*	PTS cellbiose-specific enzyme IIC	1.94	Yes
	*lmo0023*	PTS fructose-specific enzyme IIC	1.70	Yes
	*lmo2708*	PTS cellbiose-specific enzyme IIC	1.40	Yes
	*lmo0782*	PTS mannose-specific enzyme IIC	−1.21	Yes
EIID	*lmo2000*	PTS mannose-specific enzyme IID	5.00	Yes
	*lmo0024*	PTS mannose-specific enzyme IID	2.24	Yes
	*lmo0098*	PTS mannose-specific enzyme IID	1.26	Yes
	*lmo0781*	PTS mannose-specific enzyme IID	−1.15	Yes
HPr	*ptsH*	PTS Phosphocarrier protein HPr	2.35	Yes
EI	*lmo1003* (*ptsI*)	PTS Phosphotransferase system, enzyme I	1.83	Yes

### YjbH controls PrfA expression in nonstress and stress conditions

To further explore the roles of YjbH in controlling the expression of PrfA, the master virulence regulatory protein, we compared PrfA expression in the wild-type and Δ*yjbH* strains in the presence or absence of oxidative stress. Consistent with the previously determined fact that PrfA and PrfA-regulated virulence genes are normally very weakly expressed outside the host but strongly induced during intracellular infection, PrfA expression was extremely low in the wild-type strain with or without Cd^2+^ stress (Figure 4(f,g)). Surprisingly, PrfA expression was significantly induced in Δ*yjbH*, and such expression was strongly inhibited by complementing with YjbH (Figure 4(f,g)). In combination with the above findings on YjbH-mediated PTS regulation as well as the effects of PTS on PrfA activation, we suggest that YjbH functions as a cofactor that tightly controls PrfA expression in a PTS-dependent manner when the bacteria are outside the host.

## Discussion

**Figure 5.**
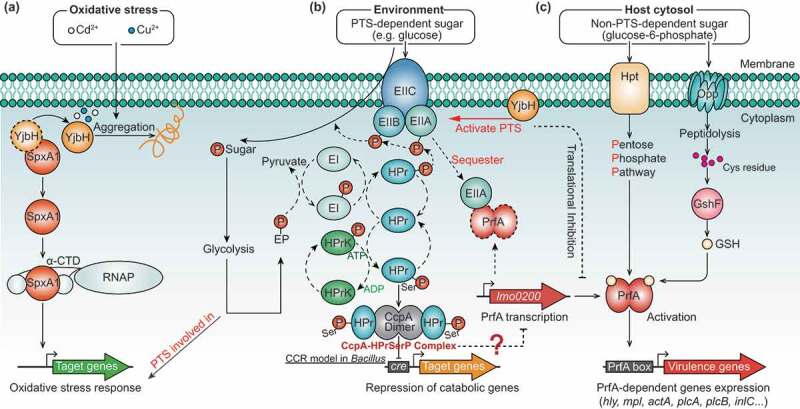
**Proposed model depicting YjbH coordinating with SpxA1, PrfA, and PTS to fine-tune the oxidative stress response and virulence of *L. monocytogenes***. (a) Localized to the plasma membrane, YjbH interacts with SpxA1, an arsenate reductase family transcriptional regulator, and contributes to defense against oxidative stress. Based on research on *B. subtills*,^26^ we speculate that under nonstress conditions, the soluble YjbH adaptor protein interacts with Spx, resulting in the rapid degradation of Spx by the ATP-dependent protease ClpXP complex. In response to stress, an aggregation of YjbH becomes surface-exposed, leading to the rapid formation of YjbH self-aggregates. (b) In the presence of PTS-dependent sugar, the PTS genes are rapidly activated by YjbH and participate in sugar transport by transferring the phosphoryl group, causing the sugar-specific EII domain A (EIIA) component of PTS to be non-phosphorylated. Non-phosphorylated EIIA has previously been proposed to bind and sequester PrfA, thereby keeping the regulator functionally inactive and preventing the expression of virulence genes.^37^ (c) In the presence of non-PTS-dependent carbon sources, the lack of PTS-dependent sugar transport results in the accumulation of phosphorylated EIIA, which is unable to sequester PrfA. Thus, the released PrfA induces the expression of the virulence genes

*L. monocytogenes* thrives in dramatically distinct environments during its transition from saprophyte to cytosolic pathogen.^37^ The intracellular lifecycle of *Listeria* requires it to survive the harsh phagosomal compartment, escape into the highly reducing cytosol, and spread to neighboring cells.^12^ Proteins in the thioredoxin family play major roles in the protection of cells against toxic oxygen species as well as maintaining the bacterial thiol–disulﬁde balance. Here, we investigated the roles of thioredoxin-like YjbH in the adaptation of *L. monocytogenes* to diverse redox environments and, more importantly, in the regulation of PTS and virulence during infection. Based mostly on studies in *Bacillus*, YjbH is known as an adaptor protein that targets Spx for ClpXP protease-mediated degradation. Here, for the first time, we demonstrated that YjbH contributes to the oxidative stress response and is able to interact with SpxA1, the Spx family member that is required for the oxidative stress response and pathogenesis of *L. monocytogenes*.^5^ Interestingly, *L. monocytogenes* YjbH was found required for the metal ion-induced oxidative stress response but not for the response to stress induced by H_2_O_2_ or the thiol-specific oxidant, diamide. Cu^2+^ and Cd^2+^ sensitivity assays are usually performed to assess oxidase and isomerase activities, respectively.^38^ As we previously determined that *L. monocytogenes* encodes a complete thioredoxin system (TrxA and TrxB) that participates in response to diamide-induced oxidative stress,^22^ we speculate that, unlike other bacterial species, *L. monocytogenes* has a delicate division of responsibilities in defending against different kinds of oxidative stress and YjbH might act as a disulﬁde bond formation protein (DSB) that has both disulfide oxidase and isomerase activities. Many Gram-positive bacteria have different complements of DSB proteins. Searches for orthologs of *B. subtilis* DsbA and DsbB (known as BdbD and BdbC, respectively) in low-G + C Gram-positive bacteria revealed that many of these organisms contain either a DsbA or DsbB protein, but not both.^39,40^ *L. monocytogenes* encodes two putative DSB proteins, Lmo0964 (i.e., YjbH) and Lmo1059, annotated as DsbA-like and DsbG, respectively, in the GenBank database. However, Lmo0964 was recently designated as a putative thioredoxin similar to *B. subtilis* YjbH and shown to contribute to expression of the ActA protein, which is required for *L. monocytogenes* actin-based motility.^11^ Based on this, together with our findings, we suggest that YjbH functions as an adaptor by interacting with Spx and also as a DSB protein, which has an essential role in the oxidative stress response and correct oxidative stress-induced protein folding in *L. monocytogenes* (Figure 5).

We found that *L. monocytogenes* YjbH can positively regulate many PTS genes under oxidative stress (in Δ*yjbH*, many PTS genes were transcriptionally downregulated, with fold changes in the hundreds). This is the first report of the regulatory relationship between YjbH and PTS in bacteria. However, we also observed that most of these PTS genes were slightly downregulated (approximately 2-fold change) in Δ*yjbH* under nonstress conditions, though these downregulations were far milder than in oxidative stress conditions. PTS is an exclusively bacterial multiprotein phosphorelay system that couples the transport of carbohydrates across the cytoplasmic membrane with their simultaneous phosphorylation, and this type of active transport is associated with bacterial resistance to oxidative stress.^41^ PTS can be induced by oxidative stress, and bacterial cells that lack a mannose PTS will have major problems in energy generation processes that are needed to launch an appropriate response to peroxide-induced stress, ultimately leading to increased peroxide sensitivity.^42,43^ In addition, in the presence of glucose, *Vibrio vulnificus* can increase pyruvate production via the interaction of the histidine protein (HPr, a component of PTS) with pyruvate kinase A (PykA) to protect against fungus-induced H_2_O_2_ stress.^44^ Therefore, we proposed a working model illustrating how YjbH may contribute to the oxidative stress response of *Listeria* by fine-tuning the activation of the PTS genes. Under oxidative stress, YjbH directly activated the PTS genes to accelerate carbohydrate uptake in order to defend against stress-induced damage. Alternatively, YjbH may indirectly regulate PTS *via* interaction with the global transcriptional regulator, Spx, although the Spx regulon in *L. monocytogenes* remains unclarified. In *B. subtilis*, oxidized Spx activates >100 redox homeostasis genes (e.g., thioredoxin, bacillithiol biosynthesis, and oxidoreductase genes), and represses approximately 170 genes involved in energy-consuming functions.^12,24,45^ Among these Spx-regulated genes, the beta-glucoside- and mannose-specific PTS components, enzyme II ABC, are directly regulated by Spx.^45^ Thus, we speculate that PTS regulation in *L. monocytogenes* is YjbH-dependent under oxidative conditions, and this regulation switches to become YjbH independent when the stress is relieved.

More importantly, we found that YjbH inhibited PrfA expression when bacteria were grown in a nutrient-rich medium with or without oxidative stress, so YjbH may function as a cofactor or adaptor that serves to tightly control PrfA expression *via* PTS during the transition of *L. monocytogenes* from outside to inside the host. PrfA activity is strongly inhibited if bacteria are grown in the presence of glucose or other PTS substrates, and repression of PrfA-dependent gene expression correlates directly with the phosphorylation status of PTS permeases (EII components) but not with phosphorylated HPr (HPr-Ser-P), which plays key roles in the induction of carbon catabolite repression (CCR).^46^ In the presence of PTS-dependent sugars, transport of these carbohydrates across the bacterial membrane results in the transfer of a phosphate group from the PTS EII domain A (EIIA) to the transported sugar and the subsequent accumulation of non-phosphorylated EIIA, which in turn serves to sequester PrfA and inhibit its activity.^47^ By contrast, the bacteria can intelligently use alternative carbon sources (like phosphorylated glucose and glycerol) during replication in the cytosol, and the pentose phosphate pathway (PPP) is the predominant sugar metabolism pathway used in host environments where expression of PrfA-dependent virulence factors is essential.^37,47,48^ However, the mechanism underlying PrfA control based on sugar availability remains unknown, though there is known to be no direct influence on PrfA activity of CCR and catabolite control protein A (CcpA), which are important for the expression of virulence genes in many pathogenic bacteria.^49-51^ Additionally, PrfA activity must be carefully modulated in response to environmental signals to enable *L. monocytogenes* to optimize bacterial fitness outside of the host. It has been previously shown that PrfA-overexpression mutants had significantly impaired growth and glucose uptake in nutrient-rich media, where glucose was the main carbon source taken up by the PTS.^52^ By contrast, PrfA-overexpression mutants exhibited a competitive advantage over the wild-type strain in media in which glycerol (a non-PTS carbon source) was the main carbon source.^53^ Therefore, the fitness defects of Δ*yjbH* in BHI media may be attributable to PrfA activation caused by the absence of YjbH. However, it is strange that we did not observe significant changes in the transcriptional levels of *prfA* in the wild-type and Δ*yjbH* strains cultured in BHI media, which suggests that there could also be post-transcriptional modification of YjbH on PrfA expression and activation. Based on these findings, we proposed a working model depicting the roles of YjbH in the control of PTS-dependent PrfA activation (Figure 5). In the presence of PTS-dependent sugars, the PTS genes are rapidly activated by YjbH and participate in sugar transport by transferring the phosphoryl group, causing the sugar-specific EIIA component of PTS to be non-phosphorylated. Non-phosphorylated EIIA has previously been proposed to bind and sequester PrfA, thereby keeping the regulator functionally inactive and preventing the expression of virulence genes.^37,54^ In the presence of non-PTS-dependent carbon sources, the lack of PTS-dependent sugar transport results in the accumulation of phosphorylated EIIA, which is unable to sequester PrfA, so the released PrfA induces virulence gene expression.^47^

In summary, for the first time, we revealed the biological roles and the underlying regulatory mechanisms of the putative thioredoxin YjbH in the oxidative stress tolerance and intracellular infection of the foodborne pathogen *L. monocytogenes*. The findings indicate that YjbH participates in regulating the key virulence genes, with a complicated regulatory network involving PrfA and PTS, and YjbH thereby contributes to bacterial stress adaption and pathogenicity. Our findings provide a valuable model for clarifying the pathways associated with the potential roles of thioredoxins from foodborne pathogens regarding improving survival in the external environment and, more importantly, successfully establishing an infection within the host.

## Materials and methods

### Bacterial strains and culture conditions

All *L. monocytogenes* strains are a derivative of wild-type EGD-e and were cultivated in Brain Heart Infusion (BHI, Oxoid), with shaking at 37°C unless otherwise indicated. All *E. coli* strains were cultivated in Luria-Bertani (LB, Oxoid) with shaking at 37°C. The antibiotics were used where appropriate with the following final concentrations: chloramphenicol (10 μg/mL), kanamycin (50 μg/mL), and tetracycline (2 μg/mL). All chemicals were purchased from Sangon Biotech, Merck, or Sigma-Aldrich and were of the highest purity available. The *L. monocytogenes* and *E. coli* strains used in this study are listed in Table S3 in Supplementary Material. All primers used in this study are listed in Table S4.

### In-frame deletion and complementation of L. monocytogenes genes

The temperature-sensitive pKSV7 shuttle plasmid was employed for allelic exchange by using the previous methods.^55^ Briefly, the constructed knock-out plasmid harboring the homologous arms upstream and downstream of the interest gene was electroporated into the competent EGD-e cells. A single colony of *L. monocytogenes* construct was grown at a non-permissive temperature (42°C) on BHI agar containing chloramphenicol to promote chromosomal integration, and after then the recombinants were successively passaged without antibiotics at a permissive temperature (30°C) for enabling plasmid excision and curing. Mutants that lost pKSV7 were identified by sensitivity to chloramphenicol, and finally, allelic exchange was confirmed by PCR and Sanger DNA sequencing when necessary.

Knock-in of pIMK2 derivative plasmids was used for complementing genes into *L. monocytogenes* by using the standard methods.^56^ Briefly, the complementation plasmid was constructed by amplifying the interest gene along with its endogenous promoter or the constitutive promoter (P_help_), and then the recombinant plasmid was electroporated into competent *L. monocytogenes* cells. Integration was confirmed by antibiotic resistance.

### Prokaryotic expression and purification

The recombinant proteins used in this study were expressed as an N-terminal His tag fusion. The full-length open reading frame (ORF) of the gene of interest was amplified and cloned into pET30a(+), and then transformed into Rosetta competent cells. *E. coli* cells harboring recombinant plasmids were grown in 500 mL LB media supplemented with 50 µg/mL kanamycin at 37°C until cultures reached 0.8–1.0 at an optical density of 600 nm (OD_600 nm_). Isopropyl β-D-1-thiogalactopyranoside (IPTG) was added to a final concentration of 0.4 mM to induce expression of recombinant proteins for an additional 3 h under optimized conditions. His-tagged fusion proteins were purified using nickel-chelated affinity column chromatography.

### Preparation of mice polyclonal antibodies

Five 6-week-old BALB/C mice were used to prepare polyclonal antibodies for each protein immunogen. Each mouse was primarily immunized *via* subcutaneous injection (0.1 mL/site, 4 sites in total) of 50 μg antigen with an equal volume of Freund’s Complete Adjuvant (Sigma). After two weeks, mice were boosted by subcutaneous injection of 50 μg antigen each in Freund’s Incomplete Adjuvant (Sigma) three times at 14-day intervals. Mice were bled ~10 days after the last injection.

### Growth curve of L. monocytogenes in BHI media

*L. monocytogenes* colonies were inoculated into BHI broth and the OD_600 nm_ of the overnight-grown cultures was measured, and the cultures were normalized to an optical density of 1.0. Normalized bacterial cultures were diluted (1:50) in fresh 25 mL BHI broth in 250-mL flasks and incubated with shaking at 37°C. Kinetic growth at OD_600 nm_ was measured every hour, 12 hours in total.

### Bacterial morphology, motility and flagellar observation

*L. monocytogenes* were grown on the BHI agar plates for 12 hours, and the bacterial colony morphology was observed by using a stereomicroscope. Motility assay was performed essentially on soft (0.25%) tryptone soya agar (TSA) according to previous methods with minor modifications.^57,58^ Specifically, overnight-grown cultures adjusted at OD_600 nm_ to 0.20 (about 2 × 10^8^ CFU/mL) and 5 μL bacterial cultures were straightly pipetted into soft TSA agar and incubated at 30°C or 37°C for 48 h to allow growth. Motility ability was assessed by examining the migration of bacteria through agar from the center toward the periphery of the colony. Furthermore, the flagellar was observed by using the transmission electron microscopy (TEM) as performed previously.^59^ Briefly, *L. monocytogenes* colonies grown overnight at 30°C from BHI agar plates were suspended in 50 μL monoethanolamine buffer (pH 10.0), and 10 μL of the suspension applied to carbon-coated copper grids and allowed to stand for 2 min at room temperature. Excess liquid was subsequently removed using filter paper, and bacteria stained with 10 μL of 0.5% phosphotungstic acid (pH 7.0) placed on the grids for 10 s at room temperature. Excess stain was gently wicked away using filter paper, and the dried grids examined under a Hitachi H-7650 transmission electron microscope.

### Oxidative stress tolerance assay

For oxidative stress, H_2_O_2_ was used as a direct oxidant and diamide as a thiol-specific oxidizing agent, while the divalent metal ions such as Cu^2+^ and Cd^2+^ were used as the redox-active stress.^60^ *L. monocytogenes* were grown overnight in BHI broth and then diluted to OD_600 nm_ of 1.0 (∼10^9^ CFU/mL) with 10 mM PBS (pH 7.4). Bacterial suspension was 10-fold serially diluted, and 10 μL of each dilution spotted onto BHI agar plates containing various concentrations of H_2_O_2_ (10–20 mM), diamide (1–2 mM), cadmium chloride (0.25–1 mM) or copper chloride (0.25–1 mM). Following incubation at 37°C for 24–48 hours, colony growth on each plate was assessed and imaged.

### Intracellular growth in murine RAW264.7 macrophages

Intracellular growth was performed accordingly on RAW264.7 macrophages. Specifically, monolayers of RAW264.7 macrophages were cultured in DMEM medium containing 20% FBS and plated in 24-well plates that contained 2 × 10^5^ cells per well. Cells were then infected with bacteria at an MOI of 0.25 for 30 min, washed twice with warmed PBS prior to replacing media, and gentamycin was added at 50 μg/mL one hour post-infection. At each time-point, three wells were added with 1 mL sterile water at 0.5, 2, 5, and 8 hours post-infection, and the lysates were 10-fold serially diluted for enumeration of viable bacteria by plating on BHI agar. Each data point represents the average of three wells.

### Plaque assay in L929 fibroblasts

The plaque assay was carried out by conventional methods. Briefly, mouse L929 fibroblasts were maintained in high-glucose DMEM medium plus FBS and 2 mM L-glutamine. Cells were plated at 1 × 10^6^ cells per well in a 6-well dish and infected at an MOI of 1:5 with *L. monocytogenes* at 37°C with 5% CO2 for 1 h. Extracellular bacteria were killed with 50 μg/mL gentamicin for an additional 1 h, and cells were washed three times with 10 mM PBS (pH 7.4) and then overlaid with 3 mL of medium plus 0.7% agarose and 10 μg/mL gentamicin. Following a 72-h incubation at 37°C, cells were fixed with 4% paraformaldehyde for 1 h and stained with crystal violet. The diameter of plaques was measured by Photoshop (Adobe) software. The plaques (50 plaques were randomly selected) size for each strain was compared to those formed by the wild-type that was set as 100%.

### Virulence assay in mice model

Infections were performed as previously described.^61^ Overnight-grown *L. monocytogenes* were diluted into PBS (10 mM, pH 7.4) to a concentration of 1 × 10^7^ CFU/mL, and 100 μL of the dilution was inoculated intraperitoneally into 8-week-old female ICR mice. For bacterial CFU recovery from organs, the mice (8 mice for each group) were euthanized at 24 and 48 h post-infection, and the livers and spleens were harvested. Organs were homogenized in PBS (10 mM, pH 7.4), and homogenates were serially diluted and then plated on BHI agar to enumerate bacterial recovery after overnight incubation. For mice survival studies, infected mice were monitored for 7 days post-infection, and survival curves were plotted using the Kaplan-Meier method, and differences in survival were determined using the Log-rank test.

### Transcriptomic profiles of L. monocytogenes exposed to oxidative stress

#### Total RNA isolation

Overnight-grown cultures of wild-type EGD-e and Δ*yjbH* mutant were transferred (1:100) into fresh BHI broth and continued to grow, shaking at 37°C until the OD_600 nm_ reached 0.6. Bacteria were collected and re-suspended in equal volumes of fresh BHI broth containing 0.25 mM Cd^2+^ and incubated statically at 37°C for an additional 1 h. The bacteria were then harvested by centrifugation, and total RNA was extracted using the Trizol method.^62^ The genomic DNA was removed using DNase I (TaKara), and RNA purity was assessed using the NanoDrop (Thermo Fisher Scientific).

#### RNA sequencing and data processing

The extracted RNA samples were sent to Mingke Biotechnology (Hangzhou) Co., Ltd., for transcriptomic sequencing by using the Illumina Hiseq2500 platform. The paired-end raw reads were trimmed and quality controlled with SeqPrep and Sickle using default parameters. After ﬁltering out low quantity sequences, clean reads were separately aligned and mapped to the reference genome with the orientation mode using HISAT and Bowtie 2 tools.^63,64^ Output data generated from sequencing were stored in the standard FASTQ format for use as inputs for subsequent analyses.

#### Differential expression analysis and functional annotation

RSEM (RNA-Seq by Expectation-Maximization)^65^ was used to quantify gene and isoform abundance. EdgeR (Empirical analysis of digital gene expression data in R)^66^ was utilized for differential expression analysis. Mapped read count normalization was applied to the data based on the number of reads per kilobase of coding sequence per million mapped reads (RPKM).^67^ The TMM (trimmed mean of M-values) method was selected to compute normalization factors and differentially expressed genes (DEGs) between two samples selected using the following criteria: (i) logarithmic of fold change greater than 1.0 and (ii) FDR (false discovery rate) less than 0.05. To determine the functions of the differentially expressed genes, the unigenes were aligned by BLASTx against the NCBI non-redundant, Swiss-Prot, KEGG, and Cluster of Orthologous Groups (COG) protein databases. GO functional enrichment analyses were carried out using Goatools and KOBAS.^68^ DEGs were significantly enriched in GO terms and metabolic pathways at Bonferroni-corrected *P*-values of less than 0.05.

### RNA purification from intracellularly grown L. monocytogenes in BMDMs

RNA extraction and purification from intracellular bacteria grown in bone marrow-derived macrophage cells (BMDMs) were carried out as previously described.^69^ BMDMs were isolated from 6–8 week-old female C57BL/6 mice and cultured in DMEM supplemented with 20% FBS, 1% L-glutamine, 1% sodium pyruvate, 14 mM 2-mercaptoethanol, and 10% 3T3 cell supernatant (from MCSF-producing 3T3 cells).^70,71^ Briefly, *L. monocytogenes* were used to infect BMDMs seeded in a 145 mm dish, resulting in a MOI of ~100. After 30 min infection, cells were washed twice with PBS to remove unattached bacteria, and fresh medium was added. At 1 h post-infection, gentamicin (50 μg/ml) was added to kill extracellular bacteria. At 6 h post-infection, intracellular bacteria were collected using 0.45 μM filter membranes and flash-freezed in liquid nitrogen. Total bacterial RNA was extracted and purified using the Trizol method and then subjected to the following transcriptional analysis.

### Real-time quantitative RT-PCR assay

To validate the reliability of transcriptome sequencing data, real-time quantification was performed using RT-qPCR with the same RNA samples used for transcriptome experiments. The specific PCR primers (listed in Table S3) were used to amplify nucleotide fragments of genes of interest between 80 and 150 bp. RT-qPCR was performed in a 20 μL reaction by using the SYBR quantitative PCR mix (TOYOBO) according to the manufacturer’s instructions. The housekeeping gene, 16SrRNA, was used as an internal control for normalization. Relative transcription levels were quantiﬁed using the 2^−ΔΔCT^ method and shown as relative fold-change.^72^

### Protein fractionation and immunoblotting

The protein fractionation and immunoblotting procedures were performed as previously described, with minor modifications.^20,73,74^ Briefly, overnight bacterial cultures were transferred 1:20 into fresh BHI, incubated for six hours shaking at 37°C, and then the bacteria were separated from the supernatant by centrifugation. **For cytoplasmic proteins**, bacterial pellet was resuspended in 1 mL extraction solution (2% Triton X-100, 1% SDS, 100 mM NaCl, 10 mM Tris-HCl, 1 mM EDTA, pH 8.0) and lysed by using the homogenizer (Bertin) at 6,000 rpm for 30 s with intermittent cooling for 30 s and then centrifuged at 12,000 g for 10 min. The pellet was discarded, and the supernatant retained as the whole-cell extract. **For secreted proteins**, the culture supernatant was treated with 10% trichloroacetic acid (TCA) on ice overnight, and the precipitated proteins were washed twice with ice-cold acetone. Washed precipitates of supernatant proteins were resuspended in SDS-PAGE sample buffer (5% SDS, 10% glycerol, 5% β-mercaptoethanol, and 50 mM Tris-HCl, pH 6.8). **For cell wall surface proteins**, bacterial pellets were resuspended in 0.5% of the original culture volume of 10 mM PBS containing 2% SDS for 30 min at 37°C with gentle shaking. Bacterial suspensions were centrifuged, and the supernatant contained the extracted cell wall proteins was applied to an 0.22 μm filter, and the filtrate was ready for use. **For membrane proteins**, the whole-cell extract was ultra-centrifuged at 100,000 g for 1 h at 4°C to obtain the membrane pellet that was then resuspended in 1 mL extraction solution and finally ultra-centrifuged at 100,000 g for an additional 1 h. The resulting supernatant fractions were removed and the pellet that represents the membrane-containing fraction were kept at −20°C before use. Separation of cytoplasmic, secreted, and cell wall fractions were verified by immunoblotting with marker proteins GAPDH, LLO,^75^ and InlB,^76^ respectively. All the protein samples were boiled and separated by SDS-PAGE. Primary antibodies were each used at a dilution of 1:2,000–5,000, including: mouse polyclonal antibody against YjbH, rabbit polyclonal antibodies against GAPDH, LLO, and InlB, and the appropriate secondary antibodies were employed according the manufacturer’s instructions. All immunoreactions were visualized using the enhanced chemiluminescence detection system (UVP Inc.).

### Coimmunoprecipitation experiments

The whole-cell lysates from *L. monocytogenes* were incubated with protein A/G plus agarose beads (CST) for 1 h at 4°C. Samples were then incubated with the rabbit polyclonal anti-SpxA1 or mouse polyclonal anti-YjbH antibodies overnight, and the protein A/G plus agarose beads were added and incubated for two additional hours. Finally, the beads were washed five times with IP buffer. Proteins were eluted and dissolved into Laemmli sample buffer containing 5% β-mercaptoethanol, incubated at 100°C for 5 min, and subjected to SDS-PAGE and immunoblotting using the rabbit polyclonal anti-SpxA1 or mouse polyclonal anti-YjbH antibodies, with the appropriate secondary antibodies, according to the method as described above.

## Supplementary Material

Supplemental Material
