# Supplementary material for: YjbH mediates the oxidative stress response and infection by regulating SpxA1 and the phosphoenolpyruvate-carbohydrate phosphotransferase system (PTS) in Listeria monocytogenes
Source: Gut Microbes. 2021 Feb 12;13(1):1884517. doi: 10.1080/19490976.2021.1884517 (PMC7889195; doi:10.1080/19490976.2021.1884517)
Supplement: Supplemental Material [file KGMI_A_1884517_SM2543.zip › Supplementary information/Table S1.docx]

**Table S1. Genes identified by transcriptome analysis as significantly up-regulated in *L. monocytogenes* Δ*yjbH* mutant.**

| **Gene name** | **Annotation** | **Fold change**  **(Δ*yjbH*/EGD-e)** | **Significance** |
| --- | --- | --- | --- |
| *lmo1429* | hypothetical protein | ∞ | Yes |
| *lmo2435* | hypothetical protein | ∞ | Yes |
| *lmo1700* | hypothetical protein | ∞ | Yes |
| *lmo0332* | hypothetical protein | ∞ | Yes |
| *lmo0693* | flagellar motor switch protein FliY | ∞ | Yes |
| *lmo2490* | CsbA protein | ∞ | Yes |
| *cheY* | chemotaxis response regulator CheY | ∞ | Yes |
| *lmo0322* | hypothetical protein | ∞ | Yes |
| *lmo2071* | hypothetical protein | ∞ | Yes |
| *lmo0694* | hypothetical protein | ∞ | Yes |
| *lmo1133* | hypothetical protein | ∞ | Yes |
| *lmo0866a* | hypothetical protein | ∞ | Yes |
| *lmo1139* | hypothetical protein | ∞ | Yes |
| *lmo1102* | cadmium efflux system accessory protein | ∞ | Yes |
| *lmo1516* | ammonium transporter NrgA | 206.98 | Yes |
| *pyrP* | uracil permease | 178.88 | Yes |
| *lmo0514* | internalin | 134.22 | Yes |
| *lmo1517* | nitrogen regulatory PII protein | 88.11 | Yes |
| *lmo2522* | cell wall-binding protein | 72.19 | Yes |
| *glnR* | glutamine synthetase repressor | 66.07 | Yes |
| *lmo1216* | N-acetylmuramoyl-L-alanine amidase | 31.33 | Yes |
| *lmo2720* | acetate-CoA ligase | 30.20 | Yes |
| *lmo0573* | hypothetical protein | 29.99 | Yes |
| *lmo0576* | hypothetical cell wall associated protein | 29.48 | Yes |
| *lmo2254* | hypothetical protein | 29.10 | Yes |
| *lmo0294* | LysR family transcriptional regulator | 22.91 | Yes |
| *lmo0586* | hypothetical protein | 20.55 | Yes |
| *pyrR* | bifunctional pyrimidine regulatory protein PyrR uracil phosphoribosyltransferase | 19.83 | Yes |
| *pyrC* | dihydroorotase | 18.92 | Yes |
| *lmo2202* | 3-oxoacyl-ACP synthase | 18.15 | Yes |
| *lmo1870* | alkaline phosphatase | 18.12 | Yes |
| *lmo1369* | phosphotransbutyrylase | 16.86 | Yes |
| *lmo0818* | cation-transporting ATPase | 16.66 | Yes |
| *lmo1067* | GTP-binding elongation factor | 16.45 | Yes |
| *lmo0732* | peptidoglycan binding protein | 15.38 | Yes |
| *glnA* | glutamine synthetase | 15.37 | Yes |
| *lmo1247* | hypothetical protein | 15.32 | Yes |
| *lmo0331* | internalin | 15.08 | Yes |
| *nadA* | quinolinate synthetase | 14.61 | Yes |
| *lmo1518* | hypothetical protein | 14.36 | Yes |
| *lmo2277* | hypothetical protein | 14.22 | Yes |
| *lmo1370* | butyrate kinase | 13.39 | Yes |
| *lmo1416* | hypothetical protein | 13.32 | Yes |
| *lmo0591* | hypothetical protein | 12.97 | Yes |
| *lmo0998* | hypothetical protein | 12.77 | Yes |
| *lmo2498* | phosphate ABC transporter permease | 12.18 | Yes |
| *lmo2437* | hypothetical protein | 12.13 | Yes |
| *lmo1211* | hypothetical protein | 11.39 | Yes |
| *lmo0585* | secreted protein | 11.36 | Yes |
| *lmo0836* | phosphate-starvation-inducible protein PsiE | 11.07 | Yes |
| *lmo2492* | hypothetical protein | 10.56 | Yes |
| *lmo1419* | hypothetical protein | 10.39 | Yes |
| *lmo1417* | hypothetical protein | 10.09 | Yes |
| *lmo0587* | secreted protein | 10.04 | Yes |
| *lmo0160* | peptidoglycan binding protein | 10.03 | Yes |
| *lmo0811* | carbonic anhydrase | 9.93 | Yes |
| *lmo0806* | transcriptional regulator | 9.68 | Yes |
| *lmo1248* | hypothetical protein | 9.61 | Yes |
| *lmo1884* | xanthine permease | 9.54 | Yes |
| *pyrAa* | carbamoyl phosphate synthase small subunit | 9.16 | Yes |
| *lmo2210* | hypothetical protein | 9.15 | Yes |
| *lmo0920* | hypothetical protein | 9.02 | Yes |
| *lmo0866* | ATP-dependent RNA helicase | 8.47 | Yes |
| *lmo0485* | hypothetical protein | 8.32 | Yes |
| *lmo0186* | hypothetical protein | 8.07 | Yes |
| *lmo0519* | multidrug resistance protein | 7.93 | Yes |
| *gbuA* | glycine/betaine ABC transporter ATP-binding protein | 7.85 | Yes |
| *lmo2427* | cell division protein FtsW | 7.75 | Yes |
| *lmo2390* | hypothetical thioredoxine reductase | 7.73 | Yes |
| *lmo1880* | RNase HI | 7.55 | Yes |
| *lmo0588* | DNA photolyase | 7.42 | Yes |
| *lmo0351* | phosphotransferase mannnose-specific family component IIA | 7.34 | Yes |
| *lmo2087* | hypothetical protein | 7.31 | Yes |
| *lmo1246* | ATP-dependent RNA helicase | 7.24 | Yes |
| *lmo1384* | hypothetical protein | 7.21 | Yes |
| *lmo0221* | Type III pantothenate kinase | 7.12 | Yes |
| *purA* | adenylosuccinate synthetase | 7.03 | Yes |
| *lmo1431* | ABC transporter ATP-binding protein | 6.99 | Yes |
| *hslO* | heat shock protein 33 | 6.90 | Yes |
| *lmo0597* | Crp/Fnr family transcriptional regulator | 6.89 | Yes |
| *lmo1500* | hypothetical protein | 6.87 | Yes |
| *lmo1640* | hypothetical protein | 6.87 | Yes |
| *lmo0590* | hypothetical protein | 6.83 | Yes |
| *lmo0771* | hypothetical protein | 6.74 | Yes |
| *lmo1622* | hypothetical protein | 6.67 | Yes |
| *lmo2352* | LysR family transcriptional regulator | 6.60 | Yes |
| *lmo0921* | hypothetical protein | 6.58 | Yes |
| *lmo2179* | peptidoglycan binding protein | 6.57 | Yes |
| *lmo0959* | undacaprenyl-phosphate N-acetylglucosaminyltransferase | 6.57 | Yes |
| *lmo1761* | sodium-dependent transporter | 6.52 | Yes |
| *lmo1688* | enoyl-ACP reductase | 6.50 | Yes |
| *lmo1243* | hypothetical protein | 6.42 | Yes |
| *lmo1300* | arsenical pump membrane protein | 6.41 | Yes |
| *lmo0411* | phosphoenolpyruvate synthase | 6.38 | Yes |
| *lmo1210* | integral membrane protein | 6.33 | Yes |
| *ilvD* | dihydroxy-acid dehydratase | 6.33 | Yes |
| *lmo1421* | osmoprotectant transport system ATP-binding protein | 6.32 | Yes |
| *lmo2686* | hypothetical protein | 6.25 | Yes |
| *lmo1515* | HTH-type transcriptional regulator cymR | 6.24 | Yes |
| *lmo0286* | transaminase | 5.96 | Yes |
| *lmo1978* | glucose-6-phosphate 1-dehydrogenase | 5.86 | Yes |
| *lmo0481* | myosin-cross-reactive antigen | 5.81 | Yes |
| *lmo1440* | hypothetical protein | 5.80 | Yes |
| *lmo1697* | hypothetical protein | 5.79 | Yes |
| *lmo2504* | cell wall-binding protein | 5.66 | Yes |
| *lmo2491* | hypothetical protein | 5.65 | Yes |
| *lmo1689* | hypothetical protein | 5.62 | Yes |
| *lmo1422* | glycine/betaine ABC transporter permease | 5.60 | Yes |
| *lmo0805* | hypothetical protein | 5.60 | Yes |
| *lmo1424* | manganese transporter | 5.58 | Yes |
| *lmo0656* | hypothetical protein | 5.58 | Yes |
| *gbuB* | glycine/betaine ABC transporter permease | 5.51 | Yes |
| *lmo0990* | hypothetical protein | 5.48 | Yes |
| *lmo1706* | transporter | 5.45 | Yes |
| *folE* | GTP cyclohydrolase I | 5.44 | Yes |
| *lmo1013* | hypothetical protein | 5.39 | Yes |
| *lmo0991* | hypothetical protein | 5.36 | Yes |
| *lmo1920* | hypothetical protein | 5.35 | Yes |
| *lmo1037* | hypothetical protein | 5.35 | Yes |
| *lmo0999* | hypothetical protein | 5.32 | Yes |
| *lmo2062* | copper transporter | 5.30 | Yes |
| *lmo0367* | hypothetical protein | 5.28 | Yes |
| *lmo0667* | ABC transporter ATP-binding protein | 5.22 | Yes |
| *lmo0559* | hypothetical protein | 5.18 | Yes |
| *lmo1418* | hypothetical protein | 5.13 | Yes |
| *lmo1438* | penicillin-binding protein | 5.13 | Yes |
| *lmo2428* | cell division protein FtsW | 5.12 | Yes |
| *lmo2713* | GW repeat-containing protein | 5.11 | Yes |
| *lmo0939* | hypothetical protein | 5.09 | Yes |
| *lmo1291* | acyltransferase | 5.05 | Yes |
| *lmo0436* | hypothetical protein | 5.01 | Yes |
| *lmo1885* | xanthine phosphoribosyltransferase | 4.94 | Yes |
| *lmo0364* | transcriptional regulator | 4.90 | Yes |
| *lmo0819* | hypothetical protein | 4.88 | Yes |
| *lmo0837* | hypothetical protein | 4.76 | Yes |
| *ctaB* | protoheme IX farnesyltransferase | 4.74 | Yes |
| *lmo1410* | hypothetical protein | 4.69 | Yes |
| *comEC* | competence protein ComEC | 4.67 | Yes |
| *murA* | UDP-N-acetylglucosamine 1-carboxyvinyltransferase | 4.66 | Yes |
| *mreB* | rod shape-determining protein MreB | 4.65 | Yes |
| *lmo1289* | internalin | 4.65 | Yes |
| *lmo2182* | ferrichrome ABC transporter ATP-binding protein | 4.64 | Yes |
| *ipk* | 4-diphosphocytidyl-2-C-methyl-D-erythritol kinase | 4.63 | Yes |
| *gbuC* | glycine/betaine ABC transporter substrate-binding protein | 4.63 | Yes |
| *lmo1296* | hypothetical protein | 4.59 | Yes |
| *lmo2588* | multidrug transporter | 4.57 | Yes |
| *lmo0764* | lipoate-protein ligase | 4.54 | Yes |
| *lmo1910* | oxidoreductase | 4.53 | Yes |
| *lmo0807* | spermidine/putrescine ABC transporter ATP-binding protein | 4.53 | Yes |
| *prs* | ribose-phosphate pyrophosphokinase | 4.51 | Yes |
| *lmo0740* | transcriptional regulator | 4.49 | Yes |
| *lmo0596* | hypothetical protein | 4.48 | Yes |
| *ilvB* | acetolactate synthase | 4.48 | Yes |
| *lmo1677* | 1,4-dihydroxy-2-naphthoate octaprenyltransferase | 4.40 | Yes |
| *lmo0272* | hypothetical protein | 4.39 | Yes |
| *hemA* | glutamyl-tRNA reductase | 4.37 | Yes |
| *pyrG* | CTP synthase | 4.36 | Yes |
| *rnpA* | ribonuclease P | 4.35 | Yes |
| *lmo0307* | hypothetical protein | 4.34 | Yes |
| *lmo0928* | 3-methyladenine DNA glycosylase | 4.33 | Yes |
| *lmo0472* | PTS fructose transporter subunit IIB | 4.32 | Yes |
| *lmo1525* | recombination protein RecJ | 4.28 | Yes |
| *greA* | transcription elongation factor GreA | 4.25 | Yes |
| *lmo1661* | hypothetical protein | 4.24 | Yes |
| *lmo0849* | amidase | 4.20 | Yes |
| *lmo1000* | phytoene dehydrogenase | 4.19 | Yes |
| *lmo2714* | pepdidoglycan bound protein | 4.16 | Yes |
| *lmo1665* | hypothetical protein | 4.12 | Yes |
| *lmo1442* | transporter | 4.10 | Yes |
| *lmo2178* | peptidoglycan binding protein | 4.04 | Yes |
| *cheA* | two-component sensor histidine kinase CheA | 4.04 | Yes |
| *lmo2042* | cell division protein MraZ | 4.03 | Yes |
| *lmo2183* | ferrichrome ABC transporter permease | 4.02 | Yes |
| *lmo1682* | transporter | 4.01 | Yes |
| *lmo2777* | multidrug transporter | 4.01 | Yes |
| *engA* | GTP-binding protein EngA | 4.00 | Yes |
| *lmo1315* | UDP pyrophosphate synthase | 4.00 | Yes |
| *lmo2128* | LacI family transcriptional regulator | 3.99 | Yes |
| *hisZ* | ATP phosphoribosyltransferase | 3.99 | Yes |
| *qoxA* | AA3-600 quinol oxidase subunit II | 3.99 | Yes |
| *lmo2690* | TetR family transcriptional regulator | 3.98 | Yes |
| *lmo1414* | acetyl-CoA:acetyltransferase | 3.97 | Yes |
| *lmo1606* | DNA translocase | 3.97 | Yes |
| *zurM* | metal (zinc) transport protein (ABC transporter, permease) | 3.95 | Yes |
| *lmo1878* | manganese transport transcriptional regulator | 3.95 | Yes |
| *lmo2688* | cell division protein FtsW | 3.94 | Yes |
| *lmo1292* | glycerophosphodiester phosphodiesterase | 3.94 | Yes |
| *lmo2377* | multidrug transporter | 3.91 | Yes |
| *lmo2070* | hypothetical protein | 3.90 | Yes |
| *lmo2826* | MFS transporter | 3.89 | Yes |
| *lmo1696* | hypothetical protein | 3.88 | Yes |
| *mreC* | rod shape-determining protein MreC | 3.85 | Yes |
| *lmo0812* | hypothetical protein | 3.84 | Yes |
| *opuCD* | glycine/betaine ABC transporter permease | 3.84 | Yes |
| *topA* | DNA topoisomerase I | 3.84 | Yes |
| *lmo2186* | hypothetical protein | 3.81 | Yes |
| *lmo0802* | hypothetical protein | 3.81 | Yes |
| *hemE* | uroporphyrinogen decarboxylase | 3.79 | Yes |
| *lmo2519* | teichoic acid linkage unit synthesis protein | 3.77 | Yes |
| *mnmA* | tRNA-specific 2-thiouridylase | 3.77 | Yes |
| *dltA* | D-alanine--poly(phosphoribitol) ligase subunit 1 | 3.76 | Yes |
| *lmo0538* | N-acyl-L-amino acid amidohydrolase | 3.69 | Yes |
| *lmo2148* | hypothetical protein | 3.69 | Yes |
| *lmo0976* | hypothetical protein | 3.68 | Yes |
| *lmo1749* | shikimate kinase | 3.67 | Yes |
| *lmo2240* | ABC transporter ATP-binding protein | 3.65 | Yes |
| *lmo2181* | hypothetical protein | 3.65 | Yes |
| *lmo0795* | hypothetical protein | 3.64 | Yes |
| *lmo1848* | metal ABC transporter permease | 3.64 | Yes |
| *lmo0430* | LysR family transcriptional regulator | 3.62 | Yes |
| *ribC* | riboflavin kinase | 3.61 | Yes |
| *lmo2687* | cell division protein FtsW | 3.60 | Yes |
| *lmo0642* | hypothetical protein | 3.59 | Yes |
| *lmo0902* | GntR family transcriptional regulator | 3.57 | Yes |
| *dltB* | DltB protein for D-alanine esterification of lipoteichoic acid and wall teichoic acid | 3.57 | Yes |
| *lmo2375* | hypothetical protein | 3.55 | Yes |
| *mraW* | S-adenosyl-methyltransferase MraW | 3.55 | Yes |
| *lmo1071* | cell division protein FtsW | 3.54 | Yes |
| *recU* | Holliday junction-specific endonuclease | 3.53 | Yes |
| *lmo2587* | hypothetical protein | 3.53 | Yes |
| *lmo0900* | hypothetical protein | 3.52 | Yes |
| *lmo0473* | hypothetical protein | 3.52 | Yes |
| *lmo1918* | hypothetical protein | 3.51 | Yes |
| *lmo1150* | transcriptional regulator PocR | 3.51 | Yes |
| *lmo1919* | hypothetical protein | 3.50 | Yes |
| *lmo0668* | ABC transporter permease | 3.50 | Yes |
| *lmo2550* | glycosyl transferase | 3.50 | Yes |
| *rho* | transcription termination factor Rho | 3.48 | Yes |
| *rpmB* | 50S ribosomal protein L28 | 3.46 | Yes |
| *lmo0757* | hypothetical protein | 3.46 | Yes |
| *lmo1843* | hypothetical protein | 3.45 | Yes |
| *murB* | UDP-N-acetylenolpyruvoylglucosamine reductase | 3.43 | Yes |
| *lmo0518* | hypothetical protein | 3.43 | Yes |
| *lmo2404* | hypothetical protein | 3.42 | Yes |
| *hisE* | phosphoribosyl-ATP pyrophosphatase | 3.42 | Yes |
| *lmo0537* | allantoate amidohydrolase | 3.41 | Yes |
| *accD* | acetyl-CoA carboxylase subunit beta | 3.41 | Yes |
| *mreD* | cell-shape determining protein MreD | 3.40 | Yes |
| *lmo0365* | hypothetical protein | 3.36 | Yes |
| *lmo0452* | hypothetical protein | 3.36 | Yes |
| *lmo2689* | magnesium-translocating P-type ATPase | 3.34 | Yes |
| *lmo0977* | hypothetical protein | 3.33 | Yes |
| *lmo1699* | chemotaxis protein | 3.32 | Yes |
| *lmo0593* | formate transporter | 3.30 | Yes |
| *lmo2460* | transcriptional regulator | 3.29 | Yes |
| *lmo0724* | hypothetical protein | 3.28 | Yes |
| *lmo1297* | aluminum resistance protein | 3.27 | Yes |
| *lmo1932* | heptaprenyl diphosphate synthase subunit I | 3.27 | Yes |
| *lmo2725* | hypothetical protein | 3.26 | Yes |
| *lmo1242* | hypothetical protein | 3.24 | Yes |
| *prfC* | peptide chain release factor 3 | 3.24 | Yes |
| *lmo0350* | hypothetical protein | 3.23 | Yes |
| *lmo1810* | fatty acid biosynthesis transcriptional regulator | 3.22 | Yes |
| *ansB* | asparagine synthetase | 3.21 | Yes |
| *lmo2497* | phosphate ABC transporter permease | 3.20 | Yes |
| *rpsR* | 30S ribosomal protein S18 | 3.19 | Yes |
| *lmo0366* | hypothetical protein | 3.19 | Yes |
| *lmo0627* | pepdidoglycan bound protein | 3.19 | Yes |
| *lmo0609* | phage shock protein E | 3.19 | Yes |
| *trmE* | RNA modification GTPase TrmE | 3.18 | Yes |
| *lmo2185* | hypothetical protein | 3.17 | Yes |
| *lmo1413* | peptidoglycan binding protein | 3.17 | Yes |
| *lmo1225* | MarR family transcriptional regulator | 3.16 | Yes |
| *rnc* | ribonuclease III | 3.16 | Yes |
| *hemN* | coproporphyrinogen III oxidase | 3.15 | Yes |
| *ctaA* | heme O oxygenase | 3.14 | Yes |
| *lmo1778* | ABC transporter ATP-binding protein | 3.14 | Yes |
| *lmo1617* | multidrug transporter | 3.13 | Yes |
| *lmo0454* | hypothetical protein | 3.13 | Yes |
| *lmo2053* | hypothetical protein | 3.12 | Yes |
| *lmo1498* | O-methyltransferase | 3.12 | Yes |
| *pnpA* | polynucleotide phosphorylase | 3.10 | Yes |
| *lmo1371* | dihydrolipoamide dehydrogenase | 3.10 | Yes |
| *lmo1337* | hypothetical protein | 3.09 | Yes |
| *lmo2495* | phosphate ABC transporter ATP-binding protein | 3.09 | Yes |
| *lmo2793* | hypothetical protein | 3.09 | Yes |
| *nusG* | transcription antitermination protein NusG | 3.09 | Yes |
| *lmo1492* | hypothetical protein | 3.09 | Yes |
| *lmo0922* | pantothenate kinase | 3.09 | Yes |
| *lmo1252* | hypothetical protein | 3.08 | Yes |
| *lmo2130* | hypothetical protein | 3.06 | Yes |
| *lmo1842* | hypothetical protein | 3.06 | Yes |
| *cobD* | threonine-phosphate decarboxylase | 3.06 | Yes |
| *lmo0756* | ABC transporter ATP-binding protein | 3.06 | Yes |
| *lmo2389* | NADH dehydrogenase | 3.06 | Yes |
| *lmo0646* | hypothetical protein | 3.03 | Yes |
| *lmo0944* | hypothetical protein | 3.03 | Yes |
| *lmo1981* | hypothetical protein | 3.03 | Yes |
| *lmo0946* | hypothetical protein | 3.02 | Yes |
| *lmo0193* | hypothetical protein | 3.00 | Yes |
| *lmo0492* | LysR family transcriptional regulator | 2.99 | Yes |
| *lmo1982* | hypothetical protein | 2.98 | Yes |
| *tgt* | queuine tRNA-ribosyltransferase | 2.95 | Yes |
| *lmo1921* | hypothetical protein | 2.94 | Yes |
| *fhuB* | queuine tRNA-ribosyltransferase | 2.93 | Yes |
| *rnhC* | ribonuclease HIII | 2.92 | Yes |
| *carB* | carbamoyl-phosphate synthetase | 2.92 | Yes |
| *lmo0601* | cell surface protein | 2.92 | Yes |
| *lmo1236* | hypothetical protein | 2.91 | Yes |
| *rpsJ* | 30S ribosomal protein S10 | 2.91 | Yes |
| *lmo2116* | hypothetical protein | 2.91 | Yes |
| *lmo1845* | hypothetical protein | 2.90 | Yes |
| *lmo0453* | hypothetical protein | 2.89 | Yes |
| *lmo1690* | hypothetical protein | 2.89 | Yes |
| *lmo1662* | hypothetical protein | 2.87 | Yes |
| *lmo1353* | hypothetical protein | 2.87 | Yes |
| *apt* | adenine phosphoribosyltransferase | 2.86 | Yes |
| *lmo1494* | 5'-methylthioadenosine/S-adenosylhomocysteine nucleosidase | 2.86 | Yes |
| *lmo0772* | transcriptional regulator | 2.85 | Yes |
| *lmo2184* | ferrichrome ABC transporter substrate-binding protein | 2.85 | Yes |
| *lmo2405* | hypothetical protein | 2.84 | Yes |
| *comEB* | competence protein ComEB | 2.84 | Yes |
| *lmo0321* | hypothetical protein | 2.83 | Yes |
| *lmo1712* | multidrug resistance protein | 2.82 | Yes |
| *lmo1979* | hypothetical protein | 2.82 | Yes |
| *lmo1751* | Uncharacterized RNA methyltransferase lmo1751 | 2.82 | Yes |
| *lmo0820* | acetyltransferase | 2.82 | Yes |
| *fbp* | fructose-1,6-bisphosphatase | 2.81 | Yes |
| *fhuG* | ferrichrome ABC transporter permease | 2.79 | Yes |
| *ubiE* | ubiquinone/menaquinone biosynthesis methyltransferase | 2.79 | Yes |
| *aroE* | 3-phosphoshikimate 1-carboxyvinyltransferase | 2.79 | Yes |
| *lmo0847* | glutamine ABC transporter | 2.79 | Yes |
| *lmo0195* | ABC transporter permease | 2.78 | Yes |
| *lmo0989* | MarR family transcriptional regulator | 2.78 | Yes |
| *atpB* | ATP synthase F0F1 subunit A | 2.77 | Yes |
| *lmo2061* | hypothetical protein | 2.77 | Yes |
| *hisF* | imidazole glycerol phosphate synthase subunit HisF | 2.76 | Yes |
| *hisG* | ATP phosphoribosyltransferase | 2.76 | Yes |
| *pbpA* | penicillin-binding protein 2A | 2.76 | Yes |
| *lmo0469* | hypothetical protein | 2.75 | Yes |
| *guaA* | GMP synthase | 2.75 | Yes |
| *lmo0666* | hypothetical protein | 2.73 | Yes |
| *opuCC* | glycine/betaine ABC transporter substrate-binding protein | 2.73 | Yes |
| *lmo0608* | ABC transporter ATP-binding protein | 2.72 | Yes |
| *lmo0821* | hypothetical protein | 2.72 | Yes |
| *lmo2845* | MFS transporter | 2.71 | Yes |
| *lmo1811* | ATP-dependent DNA helicase RecG | 2.71 | Yes |
| *lmo2416* | hypothetical protein | 2.69 | Yes |
| *lmo1074* | teichoic acid translocation permease TagG | 2.69 | Yes |
| *lmo1372* | branched-chain alpha-keto acid dehydrogenase subunit E1 | 2.67 | Yes |
| *lmo1849* | metal ABC transporter ATP-binding protein | 2.67 | Yes |
| *cdsA* | phosphatidate cytidylyltransferase | 2.67 | Yes |
| *lmo2496* | phosphate ABC transporter ATP-binding protein | 2.66 | Yes |
| *lmo0042* | DedA protein | 2.66 | Yes |
| *truB* | tRNA pseudouridine synthase B | 2.64 | Yes |
| *lmo0960* | hypothetical protein | 2.64 | Yes |
| *miaA* | tRNA delta(2)-isopentenylpyrophosphate transferase | 2.64 | Yes |
| *queA* | S-adenosylmethionine:tRNA ribosyltransferase-isomerase | 2.63 | Yes |
| *ispG* | 4-hydroxy-3-methylbut-2-en-1-yl diphosphate synthase | 2.62 | Yes |
| *lmo1077* | teichoic acid biosynthesis protein B | 2.62 | Yes |
| *lmo2378* | monovalent cation/H+ antiporter subunit A | 2.61 | Yes |
| *lmo0511* | hypothetical protein | 2.61 | Yes |
| *engB* | GTP-binding protein EngB | 2.60 | Yes |
| *lmo1714* | hypothetical protein | 2.60 | Yes |
| *lmo2259* | PTS beta-glucoside transporter subunit IIA | 2.59 | Yes |
| *gid* | tRNA (uracil-5-)-methyltransferase Gid | 2.59 | Yes |
| *lmo1959* | ferrichrome-binding protein | 2.58 | Yes |
| *lmo2754* | D-alanyl-D-alanine carboxypeptidase | 2.57 | Yes |
| *hemC* | porphobilinogen deaminase | 2.56 | Yes |
| *murC* | UDP-N-acetylmuramate--L-alanine ligase | 2.56 | Yes |
| *hisA* | 1-(5-phosphoribosyl)-5-[(5-phosphoribosylamino)methylideneamino] imidazole-4-carboxamide isomerase | 2.55 | Yes |
| *lmo2829* | nitroreductase | 2.55 | Yes |
| *lmo0101* | transcriptional regulator | 2.54 | Yes |
| *gtcA* | wall teichoic acid glycosylation protein GtcA | 2.54 | Yes |
| *metK* | methionine adenosyltransferase | 2.53 | Yes |
| *opuCB* | glycine/betaine ABC transporter permease | 2.52 | Yes |
| *lmo1076* | autolysin | 2.52 | Yes |
| *lmo2145* | hypothetical protein | 2.52 | Yes |
| *lmo0762* | ATP/GTP-binding protein | 2.52 | Yes |
| *atpE* | ATP synthase F0F1 subunit C | 2.52 | Yes |
| *lmo2100* | GntR family transcriptional regulator | 2.52 | Yes |
| *lmo0459* | transcriptional regulator | 2.51 | Yes |
| *lmo0009* | spermidine acetyltransferase | 2.50 | Yes |
| *hisB* | imidazoleglycerol-phosphate dehydratase | 2.50 | Yes |
| *purM* | phosphoribosylaminoimidazole synthetase | 2.49 | Yes |
| *trxB* | thioredoxin reductase | 2.49 | Yes |
| *lmo0995* | hypothetical protein | 2.49 | Yes |
| *lepA* | GTP-binding protein LepA | 2.49 | Yes |
| *rpsF* | 30S ribosomal protein S6 | 2.49 | Yes |
| *lmo0194* | ABC transporter, ATP-binding protein | 2.49 | Yes |
| *lmo2719* | hypothetical protein | 2.48 | Yes |
| *lmo1513* | iron-sulfur cofactor synthesis protein | 2.48 | Yes |
| *lmo0908* | hypothetical protein | 2.47 | Yes |
| *lmo0495* | hypothetical protein | 2.46 | Yes |
| *lmo0644* | hypothetical protein | 2.46 | Yes |
| *hisD* | histidinol dehydrogenase | 2.45 | Yes |
| *lmo1432* | hypothetical protein | 2.45 | Yes |
| *accA* | acetyl-CoA carboxylase carboxyltransferase subunit alpha | 2.45 | Yes |
| *lmo1064* | transporter | 2.45 | Yes |
| *lmo2355* | multidrug resistance protein | 2.45 | Yes |
| *divIB* | cell division protein FtsQ | 2.44 | Yes |
| *ilvA* | threonine dehydratase | 2.44 | Yes |
| *lmo2794* | NA-binding protein Spo0J | 2.44 | Yes |
| *lmo2751* | ABC transporter ATP-binding protein | 2.44 | Yes |
| *lmo0603* | hypothetical protein | 2.44 | Yes |
| *lmo1976* | oxidoreductase | 2.43 | Yes |
| *lmo2463* | multidrug transporter | 2.43 | Yes |
| *lmo0397* | hypothetical protein | 2.43 | Yes |
| *gidA* | tRNA uridine 5-carboxymethylaminomethyl modification enzyme GidA | 2.42 | Yes |
| *lmo1495* | hypothetical protein | 2.42 | Yes |
| *ftsE* | cell division protein FtsE | 2.42 | Yes |
| *lmo1865* | hypothetical protein | 2.41 | Yes |
| *lmo2371* | ABC transporter permease | 2.41 | Yes |
| *lmo2752* | ABC transporter ATP-binding protein | 2.40 | Yes |
| *lmo0793* | hypothetical protein | 2.40 | Yes |
| *lmo2499* | phosphate ABC transporter substrate-binding protein | 2.40 | Yes |
| *lmo2060* | hypothetical protein | 2.40 | Yes |
| *lmo1387* | pyrroline-5-carboxylate reductase | 2.40 | Yes |
| *lmo0484* | hypothetical protein | 2.40 | Yes |
| *lmo2263* | hypothetical protein | 2.38 | Yes |
| *lmo0803* | Na+/H+ antiporter | 2.38 | Yes |
| *lmo1392* | peptidase | 2.38 | Yes |
| *lmo0497* | sugar transferase | 2.38 | Yes |
| *lmo0192* | PurR family transcriptional regulator | 2.37 | Yes |
| *lmo1652* | ABC transporter ATP-binding protein | 2.37 | Yes |
| *qoxB* | AA3-600 quinol oxidase subunit I | 2.37 | Yes |
| *lmo0600* | hypothetical protein | 2.37 | Yes |
| *lmo2147* | hypothetical protein | 2.36 | Yes |
| *lmo0779* | hypothetical protein | 2.36 | Yes |
| *lmo1922* | hypothetical protein | 2.36 | Yes |
| *lmo1623* | hypothetical protein | 2.36 | Yes |
| *lmo1659* | hypothetical protein | 2.36 | Yes |
| *tyrA* | prephenate dehydrogenase | 2.35 | Yes |
| *lmo2051* | hypothetical protein | 2.35 | Yes |
| *lmo2604* | hypothetical protein | 2.35 | Yes |
| *lmo0618* | protein kinase | 2.35 | Yes |
| *lmo0100* | hypothetical protein | 2.35 | Yes |
| *lmo0664* | acetyl transferase | 2.34 | Yes |
| *lmo0111* | hypothetical protein | 2.34 | Yes |
| *rplC* | 50S ribosomal protein L3 | 2.34 | Yes |
| *hemH* | ferrochelatase | 2.32 | Yes |
| *obgE* | GTPase ObgE | 2.32 | Yes |
| *lmo2203* | N-acetylmuramoyl-L-alanine amidase | 2.32 | Yes |
| *lmo0927* | hypothetical protein | 2.31 | Yes |
| *lmo0782* | PTS mannose transporter subunit IIC | 2.31 | Yes |
| *lmo1075* | teichoic acid ABC transporter ATP-binding protein | 2.31 | Yes |
| *zurA* | metal (zinc) transport protein(ABC transporter, ATP-binding protein) | 2.31 | Yes |
| *lmo0185* | hypothetical protein | 2.30 | Yes |
| *lmo1373* | branched-chain alpha-keto acid dehydrogenase subunit E1 | 2.30 | Yes |
| *glmU* | bifunctional N-acetylglucosamine-1-phosphate uridyltransferase/glucosamine-1-phosphate acetyltransferase | 2.30 | Yes |
| *lmo1528* | hypothetical protein | 2.30 | Yes |
| *lmo0929* | sortase | 2.30 | Yes |
| *lmo2048* | hypothetical protein | 2.29 | Yes |
| *lmo0388* | hypothetical protein | 2.29 | Yes |
| *hisC* | histidinol-phosphate aminotransferase | 2.28 | Yes |
| *lmo0394* | P60 protein | 2.27 | Yes |
| *folK* | 7,8-dihydro-6-hydroxymethylpterin pyrophosphokinase | 2.26 | Yes |
| *gpsA* | NAD(P)H-dependent glycerol-3-phosphate dehydrogenase | 2.26 | Yes |
| *lmo1024* | hypothetical protein | 2.26 | Yes |
| *lmo0842* | peptidoglycan binding protein | 2.26 | Yes |
| *lmo1040* | molybdenum ABC transporter permease | 2.26 | Yes |
| *lmo1393* | peptidase | 2.24 | Yes |
| *lmo2454* | hypothetical protein | 2.24 | Yes |
| *opuCA* | glycine/betaine ABC transporter ATP-binding protein | 2.24 | Yes |
| *ssb* | single-strand binding protein | 2.24 | Yes |
| *lmo1284* | hypothetical protein | 2.23 | Yes |
| *infB* | translation initiation factor IF-2 | 2.23 | Yes |
| *lmo0831* | hypothetical protein | 2.22 | Yes |
| *lmo0781* | PTS mannose transporter subunit IID | 2.22 | Yes |
| *lmo1872* | methyltransferase | 2.22 | Yes |
| *lmo1323* | hypothetical protein | 2.21 | Yes |
| *lmo1079* | hypothetical protein | 2.21 | Yes |
| *lmo0851* | hypothetical protein | 2.20 | Yes |
| *lmo1396* | phosphatidylglycerophosphate synthase | 2.20 | Yes |
| *lmo1226* | transporter | 2.20 | Yes |
| *purN* | phosphoribosylglycinamide formyltransferase | 2.20 | Yes |
| *lmo2360* | transmembrane protein | 2.19 | Yes |
| *lmo1485* | hypothetical protein | 2.19 | Yes |
| *nusA* | transcription elongation factor NusA | 2.19 | Yes |
| *plsX* | glycerol-3-phosphate acyltransferase PlsX | 2.18 | Yes |
| *lmo1977* | hypothetical protein | 2.18 | Yes |
| *lmo0444* | hypothetical protein | 2.18 | Yes |
| *lmo0524* | sulfate transporter | 2.18 | Yes |
| *lmo2576* | colossin A | 2.18 | Yes |
| *lmo1692* | hypothetical protein | 2.17 | Yes |
| *lmo0723* | metyl-accepting chemotaxis protein | 2.17 | Yes |
| *lmo0440* | hypothetical protein | 2.16 | Yes |
| *ftsX* | cell division protein FtsX | 2.16 | Yes |
| *fhuC* | ferrichrome ABC transporter ATP-binding protein | 2.16 | Yes |
| *lmo1374* | branched-chain alpha-keto acid dehydrogenase subunit E2 | 2.16 | Yes |
| *lmo1881* | 5'-3' exonuclease | 2.15 | Yes |
| *lmo1869* | hypothetical protein | 2.15 | Yes |
| *lmo1568* | hypothetical protein | 2.15 | Yes |
| *lmo2056* | hypothetical protein | 2.15 | Yes |
| *lmo0227* | hypothetical protein | 2.15 | Yes |
| *purF* | amidophosphoribosyltransferase | 2.14 | Yes |
| *lmo2372* | ABC transporter ATP-binding protein | 2.13 | Yes |
| *lmo1698* | ribosomal-protein-alanine N-acetyltransferase | 2.13 | Yes |
| *hisH* | imidazole glycerol phosphate synthase subunit HisH | 2.13 | Yes |
| *bvrA* | transcription antiterminator | 2.13 | Yes |
| *lmo2219* | foldase | 2.12 | Yes |
| *lmo0728* | riboflavin kinase / FAD synthase | 2.11 | Yes |
| *purH* | bifunctional phosphoribosylaminoimidazolecarboxamide formyltransferase/IMP cyclohydrolase | 2.11 | Yes |
| *lmo0788* | hypothetical protein | 2.11 | Yes |
| *lmo1846* | multidrug transporter | 2.10 | Yes |
| *lmo0513* | hypothetical protein | 2.10 | Yes |
| *lmo0822* | transcriptional regulator | 2.10 | Yes |
| *lmo1450* | DEAD/DEAH box helicase | 2.10 | Yes |
| *lmo0327* | cell surface protein | 2.10 | Yes |
| *lmo2750* | para-aminobenzoate synthase subunit I | 2.10 | Yes |
| *zurR* | ZurR family transcriptional regulator | 2.10 | Yes |
| *lmo2215* | ABC transporter ATP-binding protein | 2.10 | Yes |
| *coaD* | phosphopantetheine adenylyltransferase | 2.10 | Yes |
| *lmo1636* | ABC transporter ATP-binding protein | 2.09 | Yes |
| *dapF* | diaminopimelate epimerase | 2.09 | Yes |
| *lmo1070* | hypothetical protein | 2.09 | Yes |
| *lmo1092* | nicotinate phosphoribosyltransferase | 2.08 | Yes |
| *lmo1647* | 1-acylglycerol-3-phosphate O-acyltransferase | 2.08 | Yes |
| *lmo1670* | hypothetical protein | 2.08 | Yes |
| *lmo0008* | cardiolipin synthase | 2.08 | Yes |
| *lmo1230* | hypothetical protein | 2.08 | Yes |
| *lmo2578* | hypothetical protein | 2.08 | Yes |
| *udk* | uridine kinase | 2.06 | Yes |
| *lmo2072* | redox-sensing transcriptional repressor Rex | 2.05 | Yes |
| *lmo1875* | ABC transporter ATP-binding protein | 2.05 | Yes |
| *cbiP* | cobyric acid synthase CbiP | 2.05 | Yes |
| *lmo1686* | hypothetical protein | 2.05 | Yes |
| *lmo0540* | penicillin-binding protein | 2.05 | Yes |
| *lmo1385* | hypothetical protein | 2.04 | Yes |
| *lmo0333* | internalin | 2.04 | Yes |
| *lmo2852* | hypothetical protein | 2.04 | Yes |
| *purQ* | phosphoribosylformylglycinamidine synthase II | 2.04 | Yes |
| *lmo2521* | polyglycerol phosphate biosynthesis protein TagA | 2.03 | Yes |
| *lmo0992* | hypothetical protein | 2.03 | Yes |
| *lmo0867* | hypothetical protein | 2.02 | Yes |
| *lmo2419* | ABC transporter ATP-binding protein | 2.02 | Yes |
| *lmo1713* | rod shape-determining protein MreB | 2.02 | Yes |
| *smc* | chromosome condensation protein Smc | 2.01 | Yes |
| *lmo2243* | AraC family transcriptional regulator | 2.01 | Yes |
| *hemD* | uroporphyrinogen-III synthase | 2.00 | Yes |
| *lmo2712* | gluconate kinase | 2.00 | Yes |
