# Supplementary material for: YjbH mediates the oxidative stress response and infection by regulating SpxA1 and the phosphoenolpyruvate-carbohydrate phosphotransferase system (PTS) in Listeria monocytogenes
Source: Gut Microbes. 2021 Feb 12;13(1):1884517. doi: 10.1080/19490976.2021.1884517 (PMC7889195; doi:10.1080/19490976.2021.1884517)
Supplement: Supplemental Material [file KGMI_A_1884517_SM2543.zip › Supplementary information/Table S2.docx]

**Table S2. Genes identified by transcriptome analysis as significantly down-regulated in *L. monocytogenes* Δ*yjbH* mutant.**

| **Gene name** | **Annotation** | **Fold change**  **(EGD-e/Δ*yjbH*)** | **Significance** |
| --- | --- | --- | --- |
| *lmo2782* | PTS cellbiose transporter subunit IIB | ∞ | Yes |
| *lmo0633* | PTS fructose transporter subunit IIB | ∞ | Yes |
| *lmo0964* | YjbH | ∞ | Yes |
| *lmo2326* | hypothetical protein | ∞ | Yes |
| *lmo0736* | ribose-5-phosphate isomerase B | 1843.44 | Yes |
| *lmo0737* | hypothetical protein | 1553.77 | Yes |
| *lmo2799* | PTS mannitol transporter subunit IIBC | 1236.41 | Yes |
| *lmo0735* | ribulose-5-phosphate 3-epimerase | 1204.21 | Yes |
| *lmo2800* | dehydrogenase | 1062.43 | Yes |
| *lmo2801* | N-acetylmannosamine-6-phosphate 2-epimerase | 1030.24 | Yes |
| *lmo0738* | PTS beta-glucoside transporter subunit IIABC | 923.26 | Yes |
| *lmo0739* | 6-phospho-beta-glucosidase | 593.08 | Yes |
| *lmo2765* | PTS cellbiose transporter subunit IIA | 540.58 | Yes |
| *lmo2764* | ROK family transcriptional regulator | 491.71 | Yes |
| *lmo2763* | PTS cellbiose transporter subunit IIC | 445.00 | Yes |
| *lmo2761* | beta-glucosidase | 237.70 | Yes |
| *lmo2798* | phosphatase | 221.52 | Yes |
| *lmo2121* | PTS mannitol transporter subunit IIBC | 181.40 | Yes |
| *lmo0181* | sugar ABC transporter substrate-binding protein | 172.51 | Yes |
| *lmo1997* | PTS mannose transporter subunit IIA | 147.12 | Yes |
| *lmo0477* | secreted protein | 138.98 | Yes |
| *lmo2122* | maltodextrose utilization protein MalA | 113.74 | Yes |
| *lmo2648* | phosphotriesterase | 103.50 | Yes |
| *lmo0048* | sensor histidine kinase AgrB | 101.29 | Yes |
| *lmo2797* | PTS mannitol transporter subunit IIA | 99.59 | Yes |
| *lmo2337* | DeoR family transcriptional regulator | 93.75 | Yes |
| *lmo1998* | opine catabolism protein | 90.08 | Yes |
| *lmo2646* | hypothetical protein | 87.99 | Yes |
| *lmo2647* | creatinine amidohydrolase | 87.72 | Yes |
| *fruB* | fructose-1-phosphate kinase | 81.25 | Yes |
| *lmo2644a* | hypothetical protein | 81.25 | Yes |
| *lmo2123* | sugar ABC transporter permease | 78.25 | Yes |
| *lmo0051* | response regulator | 76.95 | Yes |
| *lmo0180* | sugar ABC transporter permease | 74.54 | Yes |
| *lmo1999* | hypothetical protein | 73.21 | Yes |
| *lmo0182* | hypothetical protein | 71.65 | Yes |
| *lmo0179* | sugar ABC transporter permease | 58.26 | Yes |
| *plcB* | phospholipase C | 52.24 | Yes |
| *lmo2661* | ribulose-5-phosphate 3-epimerase | 50.46 | Yes |
| *lmo0298* | PTS beta-glucoside transporter subunit IIC | 49.04 | Yes |
| *lmo2662* | ribose 5-phosphate epimerase | 44.54 | Yes |
| *lmo2096* | PTS galacticol transporter subunit IIC | 40.70 | Yes |
| *fruA* | PTS fructose transporter subunit IIABC | 40.28 | Yes |
| *lmo0050* | histidine kinase | 39.74 | Yes |
| *lmo0300* | phospho-beta-galactosidase | 38.70 | Yes |
| *ulaA* | PTS system ascorbate transporter subunit IIC | 37.84 | Yes |
| *lmo2099* | transcriptional antiterminator | 37.54 | Yes |
| *lmo0426* | PTS fructose transporter subunit IIA | 33.54 | Yes |
| *lmo2664* | sorbitol dehydrogenase | 33.46 | Yes |
| *lmo2000* | PTS mannose transporter subunit IID | 32.11 | Yes |
| *actA* | actin-assembly inducing protein precursor | 31.85 | Yes |
| *lmo2665* | PTS galacticol transporter subunit IIC | 31.71 | Yes |
| *lmo2663* | polyol dehydrogenase | 30.44 | Yes |
| *mpl* | Zinc metalloproteinase precursor | 29.87 | Yes |
| *lmo2667* | PTS galacticol transporter subunit IIA | 29.33 | Yes |
| *lmo2098* | PTS galacticol transporter subunit IIA | 28.90 | Yes |
| *lmo2666* | PTS galacticol transporter subunit IIB | 27.57 | Yes |
| *lmo2095* | phosphofructokinase | 27.12 | Yes |
| *lmo0183* | alpha-glucosidase | 25.86 | Yes |
| *lmo2124* | sugar ABC transporter permease | 23.48 | Yes |
| *lmo0813* | fructokinase | 22.68 | Yes |
| *lmo2094* | L-fuculose-phosphate aldolase | 22.04 | Yes |
| *inlC* | internalin C | 21.89 | Yes |
| *lmo1143* | PduT protein | 21.56 | Yes |
| *lmo2660* | transketolase | 21.45 | Yes |
| *lmo2125* | sugar ABC transporter substrate-binding protein | 20.91 | Yes |
| *alsS* | acetolactate synthase | 20.48 | Yes |
| *lmo0917* | beta-glucosidase | 19.48 | Yes |
| *groES* | co-chaperonin GroES | 18.99 | Yes |
| *lmo0536* | 6-phospho-beta-glucosidase | 18.42 | Yes |
| *lmo2847* | rhamnulose-1-phosphate aldolase | 18.13 | Yes |
| *lmo0879* | hypothetical protein | 17.70 | Yes |
| *lmo0425* | transcriptional antiterminator BglG | 17.31 | Yes |
| *lmo0428* | PTS fructose transporter subunit IIC | 16.68 | Yes |
| *lmo0643* | transaldolase | 16.58 | Yes |
| *clpE* | ATP-dependent protease | 16.53 | Yes |
| *lmo0401* | alpha-mannosidase | 16.28 | Yes |
| *lmo2796* | transcriptional regulator | 16.21 | Yes |
| *lmo2650* | MFS transporter | 16.19 | Yes |
| *lmo0878* | oxidoreductase | 15.95 | Yes |
| *lmo0297* | transcriptional antiterminator BglG | 15.75 | Yes |
| *lmo2163* | oxidoreductase | 15.75 | Yes |
| *lmo2251* | amino acid ABC transporter ATP-binding protein | 15.27 | Yes |
| *plcA* | phosphatidylinositol-specific phospholipase c | 15.25 | Yes |
| *lmo0429* | sugar hydrolase | 15.11 | Yes |
| *lmo2659* | ribulose-phosphate 3-epimerase | 14.99 | Yes |
| *lmo0178* | xylose repressor | 14.84 | Yes |
| *lmo2668* | transcriptional antiterminator BglG | 14.23 | Yes |
| *lmo2252* | aspartate aminotransferase | 13.85 | Yes |
| *lmo2001* | PTS mannose transporter subunit IIC | 13.73 | Yes |
| *lmo2651* | PTS mannitol transporter subunit IIA | 13.71 | Yes |
| *lmo2781* | beta-glucosidase | 13.51 | Yes |
| *lmo0279* | anaerobic ribonucleoside triphosphate reductase | 13.31 | Yes |
| *lmo0859* | sugar ABC transporter substrate-binding protein | 13.26 | Yes |
| *lmo2102* | hypothetical protein | 13.05 | Yes |
| *lmo2848* | L-rhamnose isomerase | 12.91 | Yes |
| *lmo0130* | 5'-nucleotidase | 12.60 | Yes |
| *lmo0384* | IolB protein | 12.58 | Yes |
| *lmo0184* | oligo-1,6-glucosidase | 12.01 | Yes |
| *groEL* | molecular chaperone GroEL | 11.98 | Yes |
| *lmo0876* | PTS sugar transporter subunit IIC | 11.86 | Yes |
| *lmo2161* | hypothetical protein | 11.66 | Yes |
| *lmo2669* | hypothetical protein | 11.61 | Yes |
| *lmo0280* | anaerobic ribonucleotide reductase activator protein | 11.57 | Yes |
| *lmo1142* | PduS protein | 11.49 | Yes |
| *lmo0730* | hypothetical protein | 11.25 | Yes |
| *lmo0729* | hypothetical protein | 11.18 | Yes |
| *lmo2162* | hypothetical protein | 11.16 | Yes |
| *lmo0502* | sugar-phosphate isomerase | 10.61 | Yes |
| *lmo1254* | alpha,alpha-phosphotrehalase | 10.42 | Yes |
| *lmo0355* | fumarate reductase subunit A | 10.36 | Yes |
| *lmo0862* | oligo-1,6-glucosidase | 10.25 | Yes |
| *lmo0386* | IolD protein | 10.19 | Yes |
| *lmo2318* | hypothetical protein | 10.12 | Yes |
| *lmo2804* | hypothetical protein | 9.95 | Yes |
| *lmo0036* | putrescine carbamoyltransferase | 9.73 | Yes |
| *lmo2101* | pyridoxal biosynthesis lyase PdxS | 9.71 | Yes |
| *lmo2172* | propionate CoA-transferase | 9.43 | Yes |
| *lmo2235* | NADH oxidase | 9.32 | Yes |
| *lmo0778* | hypothetical protein | 9.25 | Yes |
| *cbiK* | cobalt chelatase | 9.11 | Yes |
| *lmo0412* | hypothetical protein | 8.94 | Yes |
| *lmo2580* | ABC transporter ATP-binding protein | 8.90 | Yes |
| *lmo0639* | transcripitonal regulator | 8.72 | Yes |
| *arpJ* | amino acid ABC transporter permease | 8.59 | Yes |
| *lmo0916* | PTS sugar transporter subunit IIA | 8.37 | Yes |
| *lmo0734* | LacI family transcriptional regulator | 8.35 | Yes |
| *lmo0056* | heat shock protein | 8.32 | Yes |
| *lmo1034* | glycerol kinase | 8.29 | Yes |
| *lmo2340* | hypothetical protein | 8.21 | Yes |
| *lmo2581* | hypothetical protein | 8.16 | Yes |
| *lmo1138* | ATP-dependent Clp protease proteolytic subunit | 8.13 | Yes |
| *lmo0918* | transcription antiterminator BglG | 8.10 | Yes |
| *lmo0517* | phosphoglycerate mutase | 8.10 | Yes |
| *hly* | listeriolysin O precursor | 8.03 | Yes |
| *clpB* | Clp protease subunit B | 7.92 | Yes |
| *lmo2691* | autolysin | 7.91 | Yes |
| *lmo0402* | transcriptional antiterminator BglG | 7.75 | Yes |
| *lmo0786* | ACP phosphodiesterase | 7.66 | Yes |
| *lmo0153* | zinc ABC transporter substrate-binding protein | 7.64 | Yes |
| *lmo1255* | PTS trehalose transporter subunit IIBC | 7.60 | Yes |
| *argC* | N-acetyl-gamma-glutamyl-phosphate reductase | 7.57 | Yes |
| *lmo0634* | tagatose 6-phosphate kinase | 7.54 | Yes |
| *lmo2160* | hypothetical protein | 7.29 | Yes |
| *lmo0503* | PTS fructose transporter subunit IIA | 7.29 | Yes |
| *argJ* | bifunctional ornithine acetyltransferase/N-acetylglutamate synthase | 7.25 | Yes |
| *lmo2126* | maltogenic amylase | 7.09 | Yes |
| *lmo0415* | endo-1,4-beta-xylanase | 7.00 | Yes |
| *lmo2104a* | hypothetical protein | 6.96 | Yes |
| *lmo2283* | protein gp20 | 6.74 | Yes |
| *dra* | deoxyribose-phosphate aldolase | 6.74 | Yes |
| *lmo2341* | sugar kinase | 6.74 | Yes |
| *lmo2002* | PTS mannose transporter subunit IIB | 6.61 | Yes |
| *lmo2213* | hypothetical protein | 6.57 | Yes |
| *lmo0863* | hypothetical protein | 6.54 | Yes |
| *lmo2288* | protein gp15 | 6.51 | Yes |
| *lmo1992* | alpha-acetolactate decarboxylase | 6.47 | Yes |
| *lmo2585* | hypothetical protein | 6.45 | Yes |
| *pflA* | pyruvate formate-lyase | 6.43 | Yes |
| *lmo2316* | site-specific DNA-methyltransferase | 6.28 | Yes |
| *cspD* | cold-shock protein | 6.28 | Yes |
| *lmo0261* | phospho-beta-glucosidase | 6.24 | Yes |
| *lmo2066* | hypothetical protein | 6.24 | Yes |
| *lmo2280* | protein gp23 | 6.23 | Yes |
| *lmo2237* | MFS transporter permease | 6.18 | Yes |
| *lmo0508* | PTS galactitol transporter subunit IIC | 6.12 | Yes |
| *lmo2135* | PTS fructose transporter subunit IIC | 6.05 | Yes |
| *lmo2819* | carboxypeptidase | 6.03 | Yes |
| *argH* | argininosuccinate lyase | 5.96 | Yes |
| *lmo2290* | protein gp13 | 5.95 | Yes |
| *lmo0038* | agmatine deiminase 1 | 5.91 | Yes |
| *lmo0903* | hypothetical protein | 5.89 | Yes |
| *lmo0240* | hypothetical protein | 5.86 | Yes |
| *lmo0027* | PTS beta-glucoside transporter subunit IIABC | 5.85 | Yes |
| *grpE* | heat shock protein GrpE | 5.83 | Yes |
| *lmo0919* | antibiotic ABC transporter ATP-binding protein | 5.83 | Yes |
| *lmo2652* | transcriptional antiterminator | 5.77 | Yes |
| *cysS* | cysteinyl-tRNA synthetase | 5.75 | Yes |
| *lmo2324* | anti-repressor | 5.66 | Yes |
| *lmo2236* | shikimate 5-dehydrogenase | 5.65 | Yes |
| *lmo0632* | PTS fructose transporter subunit IIC | 5.61 | Yes |
| *lmo0915* | PTS sugar transporter subunit IIC | 5.59 | Yes |
| *lmo0334* | hypothetical protein | 5.55 | Yes |
| *lysA* | diaminopimelate decarboxylase | 5.43 | Yes |
| *pnp* | polynucleotide phosphorylase | 5.42 | Yes |
| *lmo0354* | fatty-acid--CoA ligase | 5.34 | Yes |
| *lmo2771* | beta-glucosidase | 5.34 | Yes |
| *lmo0278* | sugar ABC transporter ATP-binding protein | 5.33 | Yes |
| *lmo2007* | hypothetical protein | 5.30 | Yes |
| *lmo2285* | protein gp18 | 5.28 | Yes |
| *dnaK* | molecular chaperone DnaK | 5.28 | Yes |
| *argG* | argininosuccinate synthase | 5.28 | Yes |
| *cadA* | cadmium resistance protein | 5.25 | Yes |
| *lmo1033* | transketolase | 5.17 | Yes |
| *lmo2295* | protein gp8 | 5.15 | Yes |
| *lmo2138* | transcriptional regulator | 5.14 | Yes |
| *lmo0400* | PTS fructose transporter subunit IIC | 5.13 | Yes |
| *lmo2008* | ABC transporter permease | 5.13 | Yes |
| *lmo1031* | hypothetical protein | 5.12 | Yes |
| *ptsH* | phosphocarrier protein HPr | 5.11 | Yes |
| *lmo1653* | cellsurface protein | 5.07 | Yes |
| *lmo0037* | amino acid transporter | 5.07 | Yes |
| *lmo0041* | hypothetical protein | 5.05 | Yes |
| *lmo1146* | hypothetical protein | 5.01 | Yes |
| *lmo2317* | hypothetical protein | 4.94 | Yes |
| *lmo2273* | hypothetical protein | 4.93 | Yes |
| *lmo2296* | phage coat protein | 4.92 | Yes |
| *lmo0719* | hypothetical protein | 4.90 | Yes |
| *lmo2287* | tape-measure | 4.84 | Yes |
| *lmo2282* | protein gp21 | 4.77 | Yes |
| *lmo2323* | hypothetical protein | 4.77 | Yes |
| *cbiG* | cobalamin biosynthesis protein CbiG | 4.76 | Yes |
| *hrcA* | heat-inducible transcription repressor | 4.75 | Yes |
| *lmo2673* | hypothetical protein | 4.74 | Yes |
| *pdhA* | pyruvate dehydrogenase subunit E1 alpha | 4.73 | Yes |
| *lmo0024* | PTS mannose transporter subunit IID | 4.73 | Yes |
| *lmo0022* | PTS fructose transporter subunit IIB | 4.72 | Yes |
| *lmo2795* | RpiR family transcription regulator | 4.71 | Yes |
| *lmo2289* | protein gp14 | 4.69 | Yes |
| *pflC* | pyruvate-formate lyase activating enzyme | 4.68 | Yes |
| *lmo2111* | nitroreductase | 4.66 | Yes |
| *lmo0241* | hypothetical protein | 4.63 | Yes |
| *lmo2159* | oxidoreductase | 4.61 | Yes |
| *lmo2292* | protein gp11 | 4.57 | Yes |
| *lmo0174* | transposase | 4.49 | Yes |
| *lmo2685* | PTS cellbiose transporter subunit IIA | 4.47 | Yes |
| *lmo1740* | amino acid ABC transporter permease | 4.40 | Yes |
| *lmo2407* | hypothetical protein | 4.39 | Yes |
| *lmo2291* | major tail shaft protein | 4.37 | Yes |
| *lmo1798* | hypothetical protein | 4.36 | Yes |
| *lmo2293* | protein gp10 | 4.34 | Yes |
| *lmo1201* | uroporphyrinogen-III methyltransferase/uroporphyrinogen-III synthase | 4.34 | Yes |
| *lmo2171* | MFS transporter | 4.31 | Yes |
| *lmo2683* | PTS cellbiose transporter subunit IIB | 4.25 | Yes |
| *lmo2783* | PTS cellbiose transporter subunit IIC | 4.23 | Yes |
| *lmo2748* | hypothetical protein | 4.20 | Yes |
| *lmo2284* | protein gp19 | 4.19 | Yes |
| *lmo0197* | regulatory protein SpoVG | 4.19 | Yes |
| *lmo2297* | scaffolding protein | 4.15 | Yes |
| *lmo1097* | hypothetical protein | 4.13 | Yes |
| *drm* | phosphopentomutase | 4.12 | Yes |
| *lmo2143* | hypothetical protein | 4.12 | Yes |
| *lmo1250* | antibiotic resistance protein | 4.11 | Yes |
| *lmo2168* | glyoxalase | 4.11 | Yes |
| *lmo1350* | glycine dehydrogenase subunit 2 | 4.10 | Yes |
| *lmo0640* | oxidoreductase | 4.05 | Yes |
| *lmo2817* | peptidase | 4.05 | Yes |
| *lmo0039* | carbamate kinase | 4.05 | Yes |
| *trpS* | tryptophanyl-tRNA synthetase | 4.05 | Yes |
| *lmo2205* | phosphoglyceromutase | 4.04 | Yes |
| *lmo0110* | lipase | 4.04 | Yes |
| *lmo2674* | ribose-5-phosphate isomerase B | 4.02 | Yes |
| *lmo2733* | PTS fructose transporter subunit IIABC | 4.00 | Yes |
| *lmo2298* | protein gp4 | 3.96 | Yes |
| *lmo2319* | hypothetical protein | 3.93 | Yes |
| *lmo0230* | hypothetical protein | 3.92 | Yes |
| *lmo2511* | hypothetical protein | 3.89 | Yes |
| *lmo2586* | formate dehydrogenase subunit alpha | 3.88 | Yes |
| *lmo0018* | beta-glucosidase | 3.87 | Yes |
| *lmo0030* | hypothetical protein | 3.87 | Yes |
| *lmo2684* | PTS cellbiose transporter subunit IIC | 3.85 | Yes |
| *ppnK* | inorganic polyphosphate/ATP-NAD kinase | 3.83 | Yes |
| *lmo2299* | portal protein | 3.80 | Yes |
| *sigB* | RNA polymerase sigma factor SigB | 3.80 | Yes |
| *aroE* | 3-phosphoshikimate 1-carboxyvinyltransferase | 3.80 | Yes |
| *lmo0768* | sugar ABC transporter substrate-binding protein | 3.79 | Yes |
| *ctc* | 50S ribosomal protein L25 | 3.78 | Yes |
| *lmo0864* | hypothetical protein | 3.73 | Yes |
| *lmo1018* | copper homeostasis protein CutC | 3.72 | Yes |
| *lmo2766* | RpiR family transcriptional regulator | 3.71 | Yes |
| *lmo0033* | endoglucanase | 3.69 | Yes |
| *lmo2286* | protein gp17 | 3.69 | Yes |
| *lmo0641* | heavy metal-transporting ATPase | 3.66 | Yes |
| *lmo2276* | hypothetical protein | 3.64 | Yes |
| *lmo0800* | hypothetical protein | 3.59 | Yes |
| *lmo0617* | hypothetical protein | 3.57 | Yes |
| *lmo1003* | phosphotransferase system enzyme I | 3.55 | Yes |
| *lmo0720* | hypothetical protein | 3.53 | Yes |
| *lmo0229* | CtsR family transcriptional regulator | 3.51 | Yes |
| *pflB* | pyruvate formate-lyase | 3.51 | Yes |
| *lmo1580* | hypothetical protein | 3.50 | Yes |
| *lmo2234* | hypothetical protein | 3.50 | Yes |
| *lmo0869* | hypothetical protein | 3.49 | Yes |
| *cysK* | cysteine synthase | 3.49 | Yes |
| *lmo2851* | AraC family transcriptional regulator | 3.48 | Yes |
| *lmo0873* | transcriptional regulator | 3.47 | Yes |
| *lmo0638* | hypothetical protein | 3.47 | Yes |
| *lmo0914* | PTS sugar transporter subunit IIB | 3.46 | Yes |
| *lmo1717* | hypothetical protein | 3.46 | Yes |
| *lmo0242* | hypothetical protein | 3.45 | Yes |
| *lmo2584* | formate dehydrogenase accessory protein | 3.45 | Yes |
| *lmo2385* | hypothetical protein | 3.44 | Yes |
| *lmo0506* | sorbitol dehydrogenase | 3.44 | Yes |
| *lmo2274* | protein gp29 | 3.43 | Yes |
| *lmo0070* | hypothetical protein | 3.40 | Yes |
| *lmo0499* | ribulose-5-phosphate 3 epimerase | 3.40 | Yes |
| *lmo0488* | LysR family transcriptional regulator | 3.39 | Yes |
| *lmo2818* | MFS transporter | 3.39 | Yes |
| *lmo0870* | hypothetical protein | 3.39 | Yes |
| *lmo0339* | hypothetical protein | 3.37 | Yes |
| *lmo2816* | MFS transporter | 3.32 | Yes |
| *lmo2170* | hypothetical protein | 3.31 | Yes |
| *lmo2821* | internalin | 3.29 | Yes |
| *lmo0303* | putaive secreted, lysin rich protein | 3.29 | Yes |
| *lmo2839* | sugar ABC transporter substrate-binding protein | 3.28 | Yes |
| *lmo0521* | 6-phospho-beta-glucosidase | 3.26 | Yes |
| *lmo0023* | PTS fructose transporter subunit IIC | 3.24 | Yes |
| *nifJ* | pyruvate-flavodoxin oxidoreductase | 3.23 | Yes |
| *lmo2357* | hypothetical protein | 3.23 | Yes |
| *lmo1227* | uracil-DNA glycosylase | 3.23 | Yes |
| *lmo0731* | hypothetical protein | 3.22 | Yes |
| *lmo0310* | hypothetical protein | 3.21 | Yes |
| *lmo0501* | transcriptional antiterminator BglG | 3.19 | Yes |
| *lmo2105* | ferrous iron transport protein B | 3.17 | Yes |
| *fmt* | methionyl-tRNA formyltransferase | 3.15 | Yes |
| *lmo2373* | PTS beta-glucoside transporter subunit IIB | 3.15 | Yes |
| *rplT* | 50S ribosomal protein L20 | 3.14 | Yes |
| *spxA* | ArsC family transcriptional regulator | 3.13 | Yes |
| *lmo1730* | sugar ABC transporter substrate-binding protein | 3.11 | Yes |
| *lmo1739* | amino acid ABC transporter ATP-binding protein | 3.11 | Yes |
| *int* | integrase | 3.10 | Yes |
| *lmo2010* | two-component response regulator | 3.09 | Yes |
| *lmo1732* | sugar ABC transporter permease | 3.08 | Yes |
| *lmo2730* | phosphatase | 3.07 | Yes |
| *lmo2133* | fructose-1,6-biphosphate aldolase type II | 3.06 | Yes |
| *fbaA* | fructose-1,6-bisphosphate aldolase | 3.06 | Yes |
| *rsbX* | indirect negative regulation of sigma B dependant gene expression (serine phosphatase) | 3.06 | Yes |
| *lmo1955* | integrase/recombinase | 3.05 | Yes |
| *lmo0865* | phosphomannomutase | 3.05 | Yes |
| *clpC* | endopeptidase Clp ATP-binding chain C | 3.04 | Yes |
| *lmo0897* | transporter | 3.03 | Yes |
| *lmo0309* | hypothetical protein | 3.03 | Yes |
| *lmo2009* | sugar ABC transporter permease | 3.03 | Yes |
| *lmo0797* | hypothetical protein | 3.02 | Yes |
| *lmo0584* | hypothetical protein | 3.02 | Yes |
| *lmo1994* | LacI family transcriptional regulator | 3.01 | Yes |
| *lmo0455* | hypothetical protein | 3.00 | Yes |
| *lmo0933* | sugar transferase | 2.99 | Yes |
| *lmo0533* | hypothetical protein | 2.99 | Yes |
| *lmo0209* | hypothetical protein | 2.98 | Yes |
| *lmo2700* | aldo/keto reductase | 2.95 | Yes |
| *lmo0625* | hypothetical protein | 2.94 | Yes |
| *priA* | primosome assembly protein PriA | 2.93 | Yes |
| *rplL* | 50S ribosomal protein L7/L12 | 2.93 | Yes |
| *lmo2469* | amino acid transporter | 2.93 | Yes |
| *lmo2739* | NAD-dependent deacetylase | 2.92 | Yes |
| *lmo2110* | mannnose-6 phospate isomerase | 2.88 | Yes |
| *lmo2731* | RpiR family transcriptional regulator | 2.88 | Yes |
| *lmo1029* | hypothetical protein | 2.88 | Yes |
| *lmo0392* | hypothetical protein | 2.87 | Yes |
| *lmo2695* | dihydroxyacetone kinase subunit DhaK | 2.86 | Yes |
| *lmo2772* | PTS beta-glucoside transporter subunit IIABC | 2.86 | Yes |
| *lmo2275* | protein gp28 | 2.86 | Yes |
| *moaC* | molybdenum cofactor biosynthesis protein MoaC | 2.85 | Yes |
| *rplI* | 50S ribosomal protein L7/L12 | 2.85 | Yes |
| *ldh* | L-lactate dehydrogenase | 2.83 | Yes |
| *lmo0122* | hypothetical protein | 2.83 | Yes |
| *lmo0553* | hypothetical protein | 2.83 | Yes |
| *lmo2221* | hypothetical protein | 2.82 | Yes |
| *fri* | non-heme iron-binding ferritin | 2.81 | Yes |
| *lmo2441* | transcriptional regulator | 2.81 | Yes |
| *lmo0796* | hypothetical protein | 2.81 | Yes |
| *lmo2440* | hypothetical protein | 2.80 | Yes |
| *lmo2151* | hypothetical protein | 2.80 | Yes |
| *lmo1112* | hypothetical protein | 2.79 | Yes |
| *lmo0393* | hypothetical protein | 2.79 | Yes |
| *PdhB* | pyruvate dehydrogenase subunit E1 beta | 2.79 | Yes |
| *panD* | aspartate alpha-decarboxylase | 2.79 | Yes |
| *lmo2196* | peptide ABC transporter substrate-binding protein | 2.79 | Yes |
| *lmo0105* | chitinase B | 2.78 | Yes |
| *lmo1965* | hypothetical protein | 2.78 | Yes |
| *lmo1477* | oxidoreductase | 2.77 | Yes |
| *lmo2406* | hypothetical protein | 2.77 | Yes |
| *lmo2822* | hypothetical protein | 2.77 | Yes |
| *lmo1220* | hypothetical protein | 2.77 | Yes |
| *lmo2303* | hypothetical protein | 2.76 | Yes |
| *lmo0132* | inosine 5-monophosphate dehydrogenase | 2.76 | Yes |
| *sigH* | RNA polymerase factor sigma-70 | 2.76 | Yes |
| *lmo0471* | hypothetical protein | 2.75 | Yes |
| *lmo0119* | hypothetical protein | 2.73 | Yes |
| *lmo2678* | XRE family transcriptional regulator | 2.73 | Yes |
| *lmo0363* | peptidase E | 2.73 | Yes |
| *lmo2696* | dihydroxyacetone kinase | 2.73 | Yes |
| *lmo2331* | hypothetical protein | 2.72 | Yes |
| *lmo0624* | hypothetical protein | 2.71 | Yes |
| *lmo2231* | hypothetical protein | 2.71 | Yes |
| *lmo2439* | hypothetical protein | 2.70 | Yes |
| *rplJ* | 50S ribosomal protein L10 | 2.69 | Yes |
| *lmo0031* | LacI family transcription regulator | 2.69 | Yes |
| *lmo0445* | transcripitonal regulator | 2.68 | Yes |
| *lmo0126* | hypothetical protein | 2.68 | Yes |
| *cspB* | cold-shock protein | 2.67 | Yes |
| *lmo1035* | PTS beta-glucoside transporter subunit IIABC | 2.67 | Yes |
| *lmo2067* | bile acid hydrolase | 2.66 | Yes |
| *lmo1731* | sugar ABC transporter permease | 2.66 | Yes |
| *lmo2434* | glutamate decarboxylase | 2.66 | Yes |
| *lmaB* | antigen B | 2.66 | Yes |
| *moaA* | molybdenum cofactor biosynthesis protein A | 2.66 | Yes |
| *lmo2152* | thioredoxin | 2.66 | Yes |
| *lmo2365* | RofA family transcriptional regulator | 2.66 | Yes |
| *lmo0319* | phospho-beta-glucosidase | 2.64 | Yes |
| *lmo2708* | PTS cellbiose transporter subunit IIC | 2.64 | Yes |
| *lmo2065* | hypothetical protein | 2.63 | Yes |
| *lmo0212* | hypothetical protein | 2.63 | Yes |
| *azi* | preprotein translocase subunit SecA [Listeria monocytogenes EGD-e] | 2.61 | Yes |
| *lmo0035* | glucosamine--fructose-6-phosphate aminotransferase | 2.61 | Yes |
| *lmo1389* | sugar ABC transporter ATP-binding protein | 2.60 | Yes |
| *lmo2433* | acetylesterase | 2.60 | Yes |
| *lmo2358* | N-acetylglucosamine-6-phosphate isomerase | 2.60 | Yes |
| *infC* | translation initiation factor IF-3 | 2.59 | Yes |
| *lmo2637* | hypothetical protein | 2.59 | Yes |
| *lmo0274* | hypothetical protein | 2.59 | Yes |
| *lmo0121* | phage tail protein | 2.59 | Yes |
| *lmo0129* | N-acetylmuramoyl-L-alanine amidase | 2.58 | Yes |
| *lmo2657* | deoxyguanosinetriphosphate triphosphohydrolase | 2.58 | Yes |
| *lmo1830* | short-chain dehydrogenase | 2.58 | Yes |
| *lmo1609* | thioredoxin | 2.58 | Yes |
| *lmo2438* | hypothetical protein | 2.56 | Yes |
| *lmo0547* | DeoR family transcriptional regulator | 2.56 | Yes |
| *lmo0283* | ABC transporter permease | 2.55 | Yes |
| *panC* | pantoate--beta-alanine ligase | 2.55 | Yes |
| *lmo2671* | hypothetical protein | 2.54 | Yes |
| *lmo2026* | peptidoglycan binding protein | 2.54 | Yes |
| *lmo0123* | hypothetical protein | 2.54 | Yes |
| *lmo0257* | hypothetical protein | 2.53 | Yes |
| *lmo2227* | ABC transporter ATP-binding protein | 2.53 | Yes |
| *lmo2540* | phosphatase | 2.52 | Yes |
| *flaA* | flagellin | 2.51 | Yes |
| *lmo2220* | 3'-5' exoribonuclease | 2.51 | Yes |
| *lmo0061* | hypothetical protein | 2.50 | Yes |
| *lmo2248* | hypothetical protein | 2.49 | Yes |
| *lmo0340* | hypothetical protein | 2.49 | Yes |
| *lmo0557* | phosphoglycerate mutase | 2.48 | Yes |
| *eutD* | phosphotransacetylase | 2.48 | Yes |
| *lmo0930* | hypothetical protein | 2.47 | Yes |
| *lmo0047* | hypothetical protein | 2.46 | Yes |
| *aroD* | 3-dehydroquinate dehydratase | 2.46 | Yes |
| *lmo2565* | hypothetical protein | 2.45 | Yes |
| *lmo1257* | hypothetical protein | 2.45 | Yes |
| *lmo0725* | peptidoglycan-binding protein | 2.43 | Yes |
| *lmo0043* | arginine deiminase | 2.42 | Yes |
| *lmo1883* | chitinase | 2.42 | Yes |
| *pepC* | aminopeptidase | 2.41 | Yes |
| *lmo1825* | pantothenate metabolism flavoprotein | 2.41 | Yes |
| *lmo2142* | hypothetical protein | 2.39 | Yes |
| *lmo2694* | lysine decarboxylase | 2.39 | Yes |
| *lmo0098* | PTS mannose transporter subunit IID | 2.39 | Yes |
| *lmo2359* | hypothetical protein | 2.39 | Yes |
| *lmo0572* | hypothetical protein | 2.38 | Yes |
| *lmo2679* | histidine kinase | 2.38 | Yes |
| *tyrS* | tyrosyl-tRNA synthetase | 2.38 | Yes |
| *lmo2028* | hypothetical protein | 2.37 | Yes |
| *lmo2820* | amino-terminal domain-containing protein | 2.37 | Yes |
| *topB* | DNA topoisomerase III | 2.37 | Yes |
| *lmaA* | antigen A | 2.36 | Yes |
| *lmo0127* | hypothetical protein | 2.36 | Yes |
| *lmo2643* | hypothetical protein | 2.36 | Yes |
| *lmo2226* | hypothetical protein | 2.35 | Yes |
| *lmo0125* | hypothetical protein | 2.35 | Yes |
| *lmo0353* | hypothetical protein | 2.35 | Yes |
| *lmo2705* | hypothetical protein | 2.34 | Yes |
| *lmo1861* | hypothetical protein | 2.34 | Yes |
| *lmo1390* | ABC transporter permease | 2.33 | Yes |
| *recN* | DNA repair protein | 2.32 | Yes |
| *lmo2108* | N-acetylglucosamine-6-phosphate deacetylase | 2.32 | Yes |
| *lmo2144* | GntR family transcriptional regulator | 2.30 | Yes |
| *lmo1349* | glycine dehydrogenase subunit 1 | 2.30 | Yes |
| *lmo2242* | O6-methylguanine-DNA methyltransferase | 2.30 | Yes |
| *lmo2541* | tRNA threonylcarbamoyladenosine biosynthesis protein | 2.29 | Yes |
| *lmo2636* | hypothetical protein | 2.29 | Yes |
| *lmo0317* | phosphomethylpyrimidine kinase | 2.28 | Yes |
| *lmo1036* | hypothetical protein | 2.27 | Yes |
| *ccpA* | catabolite control protein A | 2.26 | Yes |
| *lmo0352* | DeoR family transcriptional regulator | 2.26 | Yes |
| *lmo0337* | hypothetical protein | 2.26 | Yes |
| *lmo0231* | ATP:guanido phosphotransferase | 2.25 | Yes |
| *lmo2074* | hypothetical protein | 2.25 | Yes |
| *lmo1867* | pyruvate phosphate dikinase | 2.24 | Yes |
| *lmo0208* | hypothetical protein | 2.23 | Yes |
| *lmo1871* | phosphoglucomutase | 2.23 | Yes |
| *lmo2583* | DNA-binding response regulator | 2.22 | Yes |
| *lmo2391* | hypothetical protein | 2.22 | Yes |
| *lmo1822* | RNA-binding Sun protein | 2.22 | Yes |
| *lmo0270* | hypothetical protein | 2.21 | Yes |
| *lmo2563* | hypothetical protein | 2.20 | Yes |
| *lmo2249* | low-affinity inorganic phosphate transporter | 2.20 | Yes |
| *rsbW* | serine-protein kinase RsbW [Listeria monocytogenes EGD-e] | 2.20 | Yes |
| *lmo0520* | transcriptional regulator | 2.20 | Yes |
| *lmo2300* | terminase large subunit from bacteriophage A118 | 2.20 | Yes |
| *hisS* | histidyl-tRNA synthetase | 2.19 | Yes |
| *lmo2415* | ABC transporter ATP-binding protein | 2.18 | Yes |
| *serS* | seryl-tRNA synthetase | 2.17 | Yes |
| *lmo0816* | regulatory protein PaiA | 2.16 | Yes |
| *lmo0825* | 3-hydroxy-3-methylglutaryl-CoA reductase | 2.16 | Yes |
| *tagD* | glycerol-3-phosphate cytidylyltransferase | 2.15 | Yes |
| *prmA* | ribosomal protein L11 methyltransferase | 2.15 | Yes |
| *lmo2453* | epoxide hydrolase | 2.14 | Yes |
| *fabG* | 3-ketoacyl-ACP reductase | 2.14 | Yes |
| *rplS* | 50S ribosomal protein L19 | 2.14 | Yes |
| *pdhC* | dihydrolipoamide acetyltransferase | 2.14 | Yes |
| *lmo2414* | aminotransferase | 2.13 | Yes |
| *lmo1608* | hypothetical protein | 2.13 | Yes |
| *lmo2734* | sugar hydrolase | 2.11 | Yes |
| *lmo0271* | phospho-beta-glucosidase | 2.11 | Yes |
| *lmo0431* | acetyltransferase | 2.11 | Yes |
| *lmo0476* | oxetanocin A resistance protein OxrB | 2.11 | Yes |
| *lmo2677* | esterase | 2.11 | Yes |
| *lmo0135* | peptide ABC transporter substrate-binding protein | 2.10 | Yes |
| *lmo2149* | hypothetical protein | 2.10 | Yes |
| *lmo1306* | hypothetical protein | 2.09 | Yes |
| *lmo2043* | MFS transporter | 2.08 | Yes |
| *pfkA* | 6-phosphofructokinase | 2.08 | Yes |
| *lmo2109* | hydrolase | 2.07 | Yes |
| *lmo2387* | hypothetical protein | 2.07 | Yes |
| *lmo2789* | hypothetical protein | 2.06 | Yes |
| *lmo1019* | hypothetical protein | 2.06 | Yes |
| *lmo0305* | L-allo-threonine aldolase | 2.06 | Yes |
| *pgi* | glucose-6-phosphate isomerase | 2.05 | Yes |
| *lmo2697* | PTS mannose transporter subunit IIA | 2.05 | Yes |
| *lmo2670* | hypothetical protein | 2.05 | Yes |
| *lemA* | LemA protein | 2.04 | Yes |
| *lmo0457* | hypothetical protein | 2.04 | Yes |
| *lmo0128* | hypothetical protein | 2.03 | Yes |
| *lmo2141* | hypothetical protein | 2.03 | Yes |
| *lmo1862* | hypothetical protein | 2.01 | Yes |
| *lmo1340* | hypothetical protein | 2.01 | Yes |
| *lmo2334* | transcriptional regulator | 2.01 | Yes |
| *lmo1090* | glycosyltransferase | 2.01 | Yes |
| *lmo0721* | fibronectin-binding protein | 2.01 | Yes |
| *lmo2399* | hypothetical protein | 2.01 | Yes |
