# Supplementary material for: YjbH mediates the oxidative stress response and infection by regulating SpxA1 and the phosphoenolpyruvate-carbohydrate phosphotransferase system (PTS) in Listeria monocytogenes
Source: Gut Microbes. 2021 Feb 12;13(1):1884517. doi: 10.1080/19490976.2021.1884517 (PMC7889195; doi:10.1080/19490976.2021.1884517)
Supplement: Supplemental Material [file KGMI_A_1884517_SM2543.zip › Supplementary information/Table S3.docx]

Table S3. *L. monocytogenes* and *E. coli* strains used in this study.

| **Strains** | **Descriptions** | **Reference or source** |
| --- | --- | --- |
| EGD-e | The wild-type strain | This study |
| 1218# | Δ*lmo0964* of the background of EGD-e | This study |
| 1067# | 1218# + pIMK2/CΔ*yjbH*_P*_yjbH_* | This study |
| 129# | 1218# + pIMK2/CΔ*yjbH*_P*_help_* | This study |
| 1139# | 1218# + pIMK2/CΔ*yjbH*_P*_dltA_* | This study |
| DH5α | For vector construction | This study |
| Rosetta | For vector expression | This study |
| 1025# | DH5α/pET30a/SpxA1 | This study |
| 1029# | DH5α/pET30a/SpxA2 | This study |
| 1036# | Rosetta/pET30a/SpxA1 | This study |
| 1045# | Rosetta/pET30a/SpxA2 | This study |
| 109# | DH5α/pET32a/YjbH | This study |
| 110# | Rosetta/pET32a/YjbH | This study |
| 1063# | DH5α/pKSV7/Δ*yjbH* | This study |
| 165# | DH5α/pIMK2/CΔ*yjbH*_P*_yjbH_* | This study |
| 125# | DH5α/pIMK2/CΔ*yjbH*_P_help_ | This study |
| 1138# | DH5α/pIMK2/CΔ*yjbH*_P*_dltA_* | This study |
