# Supplementary material for: YjbH mediates the oxidative stress response and infection by regulating SpxA1 and the phosphoenolpyruvate-carbohydrate phosphotransferase system (PTS) in Listeria monocytogenes
Source: Gut Microbes. 2021 Feb 12;13(1):1884517. doi: 10.1080/19490976.2021.1884517 (PMC7889195; doi:10.1080/19490976.2021.1884517)
Supplement: Supplemental Material [file KGMI_A_1884517_SM2543.zip › Supplementary information/Table S4.docx]

**Table S4. The PCR primers used in this study.** The nucleotides introduced to create restriction enzyme sites are underlined.

| **Primer names** | **Primer sequences (5'-3')** | **Products (bp)** | **Descriptions** | |
| --- | --- | --- | --- | --- |
| Δ*yjbH*-a | CGGGGTACCCATGAAGACTTACAAGTTTTCGGTTCACTC | 528 | | Used for construction of *yjbH* null mutant |
| Δ*yjbH*-b | TAAGTTTCCGATGTATAAATTTTGGTTAATCATTTGCTATCACCTG |  |  |  |
| Δ*yjbH*-c | ATTAACCAAAATTTATACATCGGAAACTTATAAAAAAGAAGCACC | 528 | |  |
| Δ*yjbH*-d | GCGCTGCAGTTCGGTTATCAACGATCGCCATT |  |  |  |
| YjbH-exp-fwd | CGGGGTACCATTAACCAAAATTTATATTATCAGTCAGTAGCAAATTCC | 833 | | Used for overexpression of the recombinant *yjbH* in *E. coli* |
| YjbH-exp-rev | CGCGGATCCATTAAGTTTCCGATGTATTTCCAGTGAGACTGT |  |  |  |
| SpxA1-exp-fwd | GGAATTCCATATGGTAACGTTATACACTTCACCTAGTTGCAC | 403 | | Used for overexpression of the recombinant *yjbH* in *E. coli* |
| SpxA1-exp-rev | CCGCTCGAGGTTAACCATTTTTTGCGCTTCACGTAG |  |  |  |
| CΔ*yjbH*_P*_dltA_*-a | CGCGGATCCGTAATGTGCAAACCGTTATTTACCATTTCTC | 554 | | Used for complementation of the *yjbH* deletion mutant under *dltA* promoter |
| CΔ*yjbH*_P*_dltA_*-b | ATTTTGGTTAATCATTATTGATATTCCCATTTTTAACAGTCCTTTCTTC |  |  |  |
| CΔ*yjbH*_P*_dltA_*-c | ATGGGAATATCAATAATGATTAACCAAAATTTATATTATCAGTCAGTAGCAA | 858 | |  |
| CΔ*yjbH*_P*_dltA_*-d | ACGCGTCGACTTAGTGGTGATGGTGATGATGTAAGTTTCCGATGTATTTCCAGTGAGACTGT |  |  |  |
| CΔ*yjbH*_P*_help_*-fwd | AAAACTGCAGATGATTAACCAAAATTTATATTATCAGTCAGTAGCAAATTCCA | 817 | | Used for complementation of the *yjbH* deletion mutant under *help* promoter |
| CΔ*yjbH*_P*_help_*-rev | CGGGGTACCTTATAAGTTTCCGATGTATTTCCAGTGAGACTGT |  |  |  |
| CΔ*yjbH*_P*_yjbH_*-fwd | AGCGAGCTCAAAATAATAAGTAAAAATAGATACTTTTATAGCAAAAAGACACAAATTCT | 1083 | | Used for complementation of the *yjbH* deletion mutant under its native promoter |
| CΔ*yjbH*_P*_yjbH_*-rev | CGCGGATCCTTATAAGTTTCCGATGTATTTCCAGTGAGACTG |  |  |  |
| *fruA*-RT-fwd | TCGGCTTCATGCTTCCAGTATTCG | 138 | | Used for RT-PCR |
| *fruA*-RT-rev | AACCACCAACAAGTGCTCCAAGG |  |  |  |
| *lmo0027*-RT-fwd | GCTGAAGGTGACGAAGGAAGTAGTG | 147 | |  |
| *lmo0027*-RT-rev | TGCTACAAACATTGCCGTGAAACC |  |  |  |
| *lmo0298*-RT-fwd | GACACAACAGCCATCCAA | 119 | |  |
| *lmo0298*-RT-rev | ACACCGAAGAACCAGAATAG |  |  |  |
| *lmo0400*-RT-fwd | ATGTCGCAATCTGTATTCCA | 285 | |  |
| *lmo0400*-RT-rev | AGCCGTTACCAATAGTGAAG |  |  |  |
| *lmo0402*-RT-fwd | AGGCTTGTTGAACCGCATTGAGT | 150 | |  |
| *lmo0402*-RT-rev | TTGATGTGCTCCGTCATTGGGTTT |  |  |  |
| *lmo0428*-RT-fwd | GGGCTTGTTGGGATTAGTGAAGGG | 91 | |  |
| *lmo0428*-RT-rev | CGACAGCAGAACCAAGAACCGTAG |  |  |  |
| *lmo0503*-RT-fwd | CCAGTTGTGGCTGTACGAATACTCA | 122 | |  |
| *lmo0503*-RT-rev | AGTTGTTCATCAGCACTCCTTAGCG |  |  |  |
| *lmo0508*-RT-fwd | GGACTTGCCGCCATTGCTCAA | 112 | |  |
| *lmo0508*-RT-rev | GTCCGTCCATTAGAAGCGAAACCAT |  |  |  |
| *lmo0632*-RT-fwd | TTGGTTTAGGCGTAGCAACACTTCT | 146 | |  |
| *lmo0632*-RT-rev | GATGACCCGTAATGGATCTGCTGTT |  |  |  |
| *lmo0633*-RT-fwd | AGTAACTTCATGTCCAAGCGGTGTG | 120 | |  |
| *lmo0633*-RT-rev | TCGATACCAGAGGAGCCTTGTGTT |  |  |  |
| *lmo0738*-RT-fwd | GTGGTTCACTGTGCTACACGACTT | 149 | |  |
| *lmo0738*-RT-rev | ACATCTCCAACGGTATTGCCAATGA |  |  |  |
| *lmo0876*-RT-fwd | ACGCAGCAGCACTTAACTTACCG | 140 | |  |
| *lmo0876*-RT-rev | TGTCACCATAACAGCAGCCATCAC |  |  |  |
| *lmo0915*-RT-fwd | TCTTATCCAGGCTTTGTGCTTTCGG | 80 | |  |
| *lmo0915*-RT-rev | GGCATCATAACCCACCCAGAAATCC |  |  |  |
| *lmo0916*-RT-fwd | ACTAGAGTTTAACGAAGCCGAGGAA | 138 | |  |
| *lmo0916*-RT-rev | GCATGACAGAACAGCGTGGAATAAC |  |  |  |
| *lmo0918*-RT-fwd | ACCCTCGTCGCCATCAGCAA | 92 | |  |
| *lmo0918*-RT-rev | TTGGTGTTGATTGGTTCGGAGACAA |  |  |  |
| *lmo1255*-RT-fwd | TGCTCTCGTACCTACTGTTCTTGCT | 129 | |  |
| *lmo1255*-RT-rev | AGTGCGGCGAATAACCAACTTAGTC |  |  |  |
| *lmo1997*-RT-fwd | ATTAGAAGCGTTCCTCAGTCGTGAA | 85 | |  |
| *lmo1997*-RT-rev | ATAAATCCCTAACCGCCCAGTTTCG |  |  |  |
| *lmo2000*-RT-fwd | GGCTGTTGTTGGTGCGATGGT | 141 | |  |
| *lmo2000*-RT-rev | AAGAAGGTGAAGCCAAGTGCTAGAA |  |  |  |
| *lmo2001*-RT-fwd | AAGCACAGCCGTAGCGTTAGC | 147 | |  |
| *lmo2001*-RT-rev | CGACTAATCCCTCGTGAATTTCCCT |  |  |  |
| *lmo2002*-RT-fwd | TTGTCGAATCGGTTGAAGACGCTTA | 92 | |  |
| *lmo2002*-RT-rev | TTCTCTTTGCTTCGTTCCGCCTAG |  |  |  |
| *lmo2096*-RT-fwd | TTGCTTGCTTCTGCGATTGGTTTG | 84 | |  |
| *lmo2096*-RT-rev | GCTCGCCTGCTCCGTATCAATAC |  |  |  |
| *lmo2098*-RT-fwd | GAAGCGGTAACAAGCCGAGAAGTA | 84 | |  |
| *lmo2098*-RT-rev | CGTGTCTGTATGAGGAATTGCGATT |  |  |  |
| *lmo2135*-RT-fwd | CAGTCGGCGGAGGTCTTAGTATGA | 149 | |  |
| *lmo2135*-RT-rev | GATGAGTAACATGGCTGCTGTGACA |  |  |  |
| *lmo2650*-RT-fwd | GCTGCTCGCTCAATGAATGTGGATA | 93 | |  |
| *lmo2650*-RT-rev | GACGATGACTACTTTGGCGCTTGT |  |  |  |
| *lmo2651*-RT-fwd | AAGCAATCGTGCAAGCAGGAGAA | 147 | |  |
| *lmo2651*-RT-rev | CCGTTGGTCTCGCATGAGGAATT |  |  |  |
| *lmo2665*-RT-fwd | TCGTAACTGGTGGTAACTGGTGGT | 149 | |  |
| *lmo2665*-RT-rev | TGCTGCTGTAGAACCTGTTGGTAAG |  |  |  |
| *lmo2666*-RT-fwd | TTGGTAGCTTGTGGAGCAGGAATTG | 132 | |  |
| *lmo2666*-RT-rev | GTCTTGCATGGATGCTGCTTCTGA |  |  |  |
| *lmo2667*-RT-fwd | AATGGTCGCAGAAAGTGGGAAGTC | 122 | |  |
| *lmo2667*-RT-rev | GTAATGCCACGCCGTATCCATCTAA |  |  |  |
| *lmo2668*-RT-fwd | GAATATGCCAGAAGCGGAGCGAAT | 115 | |  |
| *lmo2668*-RT-rev | TTCCAGTGCCAGCCTTAACTTGTG |  |  |  |
| *lmo2763*-RT-fwd | CGCCTATCAAGTGCCTTGGACAA | 95 | |  |
| *lmo2763*-RT-rev | GACAGAGCAAGCCGACCAGAAG |  |  |  |
| *lmo2765*-RT-fwd | CGGGCACCACGAGCATCATAAG | 137 | |  |
| *lmo2765*-RT-rev | GCACGAACTCAGTGACAACTTCTCT |  |  |  |
| *lmo2772*-RT-fwd | AAATTGGCGTAAAGCTGATGCGATT | 97 | |  |
| *lmo2772*-RT-rev | TGCCACCGATACATTATCACCAACT |  |  |  |
| *lmo2797*-RT-fwd | CAACATTGGCTGATGGAGTGGCT | 121 | |  |
| *lmo2797*-RT-rev | ACTTCTGCAAGCTCTGACAACACTT |  |  |  |
| *lmo2799*-RT-fwd | TGTTGGTGTAGCTTCTCCTGGTAGT | 124 | |  |
| *lmo2799*-RT-rev | TGGAATGGCGGTAATGAATGTGACA |  |  |  |
| *ulaA*-RT-fwd | GCCGTAACACTTGCGATGACACT | 144 | |  |
| *ulaA*-RT-rev | GCCAACAGCTTGCACAGTAATTCC |  |  |  |
| *lmo0425*-RT-fwd | TGGATGTTGCGACGATGGAAGAAG | 82 | |  |
| *lmo0425*-RT-rev | ACGATGCCAGGCTTAGCAATACC |  |  |  |
| *lmo0426-*RT-fwd | GAACGAGAAGCACATTCAACTACCG | 136 | |  |
| *lmo0426-*RT-rev | CGTCATCCAGCGATTCCCATTCAA |  |  |  |
| *lmo2099-*RT-fwd | CGAATTACGGAAGAGCAGGTTCACT | 105 | |  |
| *lmo2099*-RT-rev | AGCATCCACCGTTCCTCGTTCTA |  |  |  |
| *ptsH*-RT-fwd | GGCAACTCTATTGGTGCAGGCT | 108 | |  |
| *ptsH*-RT-rev | CCAAGAGACATAACGCCCATGATTG |  |  |  |
| *plcB*-RT-fwd | ATCATACCCTCCAGGCTACCA | 145 | |  |
| *plcB*-RT-rev | CGCCCTTTTCGCATTTTC |  |  |  |
| *actA*-RT-fwd | CAGCAGATGAGTCTTCACCACA | 92 | |  |
| *actA*-RT-rev | CCATTTCCCCGCATCTTTTA |  |  |  |
| *mpl*-RT-fwd | CAGCAAGGACAGCTTAGGATTAC | 127 | |  |
| *mpl*-RT-rev | CTTTCACTGGGTTTCCGACATA |  |  |  |
| *inlC*-RT-fwd | GAGTATTCAACGACCAACGCCTAT | 98 | |  |
| *inlC*-RT-rev | GGTCTGTAACACTTTGCTTCCCTA |  |  |  |
| *plcA*-RT-fwd | CGAGCAAAACAGCAACGATAG | 94 | |  |
| *plcA*-RT-rev | CGTGTCAGTTCTGGGAGTAGTGTAA |  |  |  |
| *hly*-RT-fwd | TCACATCGTCCATCTATTTGCC | 97 | |  |
| *hly*-RT-rev | ATTACCGTTCTCCACCATTCC |  |  |  |
| *prfA*-RT-fwd | TGCGGTCAACTTTTAATCCTG | 112 | |  |
| *prfA*-RT-rev | CGATGCCACTTGAATATCCTAACT |  |  |  |
